# Role of glucuronoxylomannan and steryl glucosides in protecting against cryptococcosis

**DOI:** 10.1128/mbio.00984-25

**Published:** 2025-04-29

**Authors:** Gabriel Soares Matos, Samyr M. Querobino, Veronica S. Brauer, Luna S. Joffe, Nivea Pereira de Sa, Caroline Mota Fernandes, Deveney DaSilva, Vanessa A. da Silva, Marinaldo Pacífico Cavalcanti Neto, Tyler Normile, Hengwei Zhu, Surita R. Bhatia, Li Tan, Parastoo Azadi, Christian Heiss, Tamara L. Doering, Maurizio Del Poeta

**Affiliations:** 1 Department of Microbiology and Immunology, Stony Brook University 273107, Stony Brook, New York, USA; 2 Department of Chemistry, Stony Brook University 603074, Stony Brook, New York, USA; 3 Complex Carbohydrate Research Center, University of Georgia 123423, Athens, Georgia, USA; 4 Department of Molecular Microbiology, Washington University School of Medicine 169015, St. Louis, Missouri, USA; 5 Division of Infectious Diseases, School of Medicine, Stony Brook University 12301, Stony Brook, New York, USA; 6 Veterans Affairs Medical Center 20077, Northport, New York, USA; Duke University Hospital, Durham, North Carolina, USA

**Keywords:** steryl glucosides, glucuronoxylomannan, xylose, mannose, glucuronic acid, *Cryptococcus neoformans*, fungal infection, vaccine, immunity

## Abstract

The development of vaccines for fungal diseases, including cryptococcosis, is an emergent line of research and development. In previous studies, we showed that a *Cryptococcus* mutant lacking the *SGL1* gene (*∆sgl1*) accumulates certain glycolipids called steryl glucosides (SGs) on the fungal capsule, promoting an effective immunostimulation that totally protects the host from a secondary cryptococcal infection. However, this protection is lost when the cryptococcal capsule is absent in the *∆sgl1* background. The cryptococcal capsule is mainly composed of glucuronoxylomannan (GXM), a polysaccharide microfiber consisting of glucuronic acid, xylose, and mannose linked by glycosidic bonds forming specific triads. In this study, we engineered cells to lack each of the GXM components and tested the effect of these deletions on protection under the condition of SG accumulation. We found that glucuronic acid and xylose are required for protection, and their absence abrogates the production of IFNγ and IL-17A by γδ T cells, which are necessary stimulants for the protective phenotype of the *∆sgl1*. We analyzed the structure of the GXM microfibers and found that although the deletion of *SGL1* only slightly affects the size and distribution of these microfibers, it significantly changes the ratio of mannose to other components. In conclusion, this study identifies the structural modifications that the deletion of *SGL1* and the consequent accumulation of SGs impart to the GXM structure of *C. neoformans*. This provides significant insights into the protective mechanisms mediated by SG accumulation on the capsule, with important implications for the future development of an efficacious cryptococcal vaccine.

**IMPORTANCE:**

*Cryptococcus neoformans* is an encapsulated fungus that causes invasive fungal infections with high morbidity and mortality in susceptible patients. With increasing drug resistance and high toxicity of current antifungal drugs, there is a need for alternative therapeutic strategies, such as a cryptococcal vaccine. In this study, we identify the necessary capsular components and their structural organization required for a cryptococcal vaccine to protect the host against challenge with a virulent strain. These capsular components are glucuronic acid, xylose, and mannose, and they work together with certain glycolipids called steryl glucosides (SGs) to stimulate host immunity. Interestingly, SGs on the capsule may favor the formation of small capsular microfibers organized in specific mannose triads. Thus, the results of this paper are important because they identify a mechanism by which SGs affect the structure of the cryptococcal capsule, with important implications for the future development of a cryptococcal vaccine using capsular components and SGs.

## INTRODUCTION

Cryptococcosis is a life-threatening fungal infection that is considered a global health threat, causing significant morbidity and mortality worldwide (1, 2). It is especially prevalent in sub-Saharan Africa (3) and the Pacific Northwest of the United States (4), infecting hundreds of thousands of people each year (5). Immunocompromised individuals, such as those with HIV/AIDS, chemotherapy patients, and organ transplant recipients, are at a high risk of infection and mortality (6, 7). If the infection is not controlled, it disseminates into the central nervous system, leading to lethal meningoencephalitis (8). With an increase in immunodeficient subjects, there has been a rise in life-threatening fungal infections (9). Current antifungal therapeutics are inadequate due to high toxicity, increasing the need for alternative therapeutic and/or preventive strategies, such as the development of an effective vaccine. Despite notable scientific and economic challenges, a cryptococcal vaccine may be a commercially viable option (10). Several investigators are working in this area (11–17), focusing on whole-cell vaccines (either live or heat-killed) or subunit vaccines using various cryptococcal components (reviewed in (18).

Recently, we demonstrated that deletion of the sterylglucosidase gene (*SGL1*) in *Cryptococcus neoformans* leads to a dramatic accumulation of the Sgl1 substrates, called steryl glucosides (SGs) (19). As these glycolipids cannot be metabolized, they accumulate within the cells, and particularly on the surface of the fungus, where the capsule is located. They are also found in the culture medium (19), but it is not known whether they are actively transported/secreted or if they are simply shed with the capsular material. The mutant that accumulates SGs, ∆*sgl1*, not only is avirulent but is eliminated from the host lung within 2 weeks. Intriguingly, if mice are subsequently challenged with a virulent *C. neoformans* wild-type strain, they are totally protected, regardless of whether the ∆*sgl1* mutant vaccine was administered dead or alive (20–22). Furthermore, the ∆*sgl1* vaccine strain is still protective even when given to immunocompromised mice lacking CD4+ T cells, CD8+ T cells, macrophages, monocytes, or neutrophils (22). However, to be protective, the ∆*sgl1* strain requires the cryptococcal capsule because a double mutant lacking the capsule (∆*cap59/*∆*sgl1*) does not confer any protection even though SGs accumulate (23). This suggests that a capsular component(s) is required for stimulating protective immunity in the presence of SGs.

**Fig 1:**
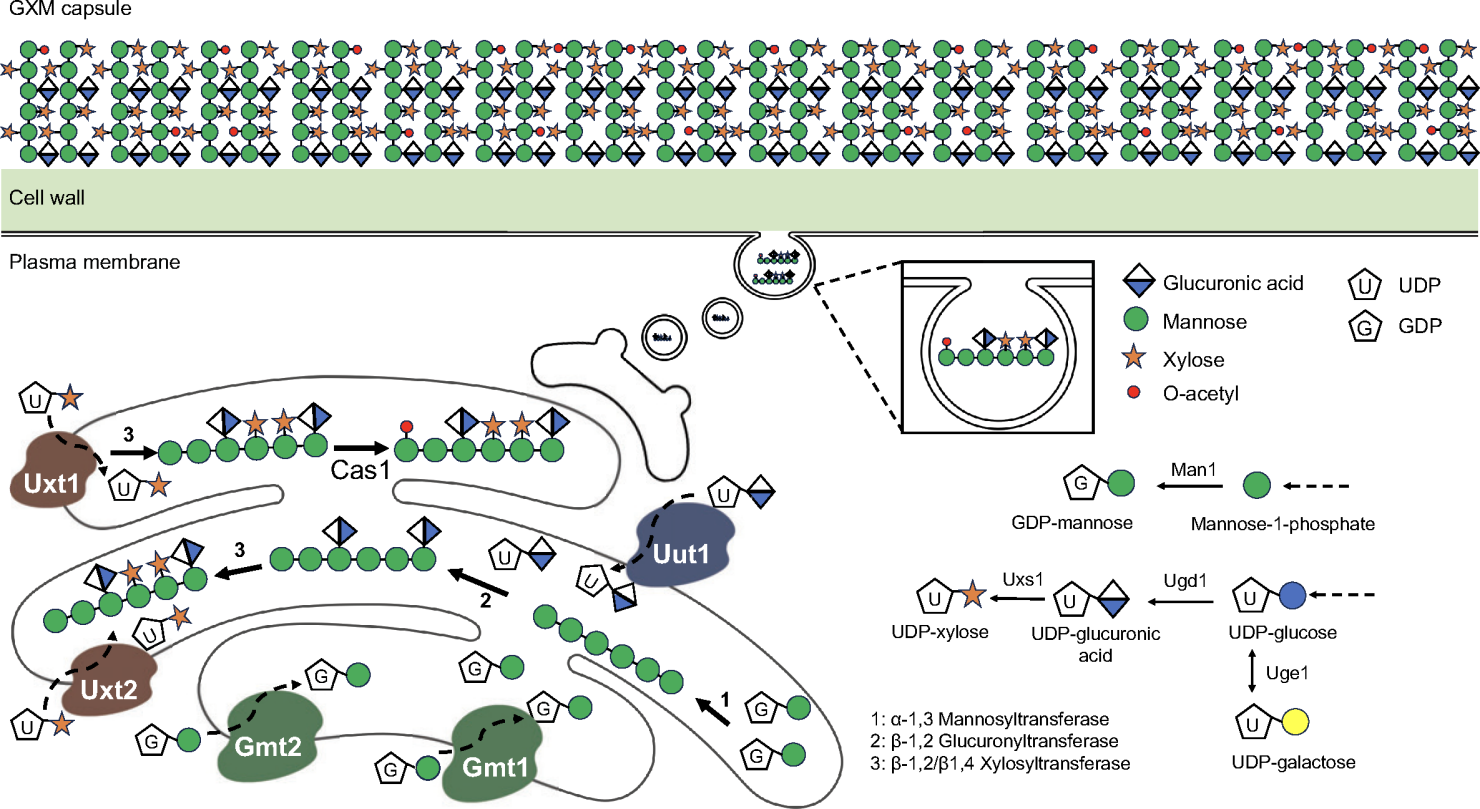
Diagram of the polysaccharide capsule of *C. neoformans*. GXM is made by sugar polymerization into an elongated carbohydrate backbone, from UDP-glucuronic acid, UDP-xylose, and GDP-mannose precursors. Precursor synthesis occurs in the cytoplasm. As shown at the lower right, GDP-mannose is produced through the activity of Man1, a phosphomannose isomerase. UDP-glucose is converted to UDP-glucuronic acid by UDP-glucose dehydrogenase (Ugd1) and then to UDP-xylose by Uxs1 decarboxylase. UDP-glucose can also be converted to UDP-galactose, which is required for GXMGal synthesis, in a reaction catalyzed by Uge1 epimerase. As shown at the bottom left, nucleotide sugars are then transported into the Golgi by Gmt1 and Gmt2 (GDP-mannose), Uxt1 and Uxt2 (UDP-xylose), and Uut1 (UDP-glucuronic acid). Mannosylation is performed by an α−1,3 mannosyltransferase (1), and the formation of a β−1,2 glycosidic bond between glucuronic acid and mannose is catalyzed by β−1,2 glucuronyltransferase (2); xylosyl can be added by β−1,2 and/or β−1,4 xylosyltransferase activities (3). O-acetyl residues are also added to the growing polysaccharide by Cas1. This sequence of events is putative. The number of xylose side chains and the amount of 6-O-acetylation varies with strain and growth conditions. After synthesis, GXM molecules exit the cell and associate with the cell wall.

The cryptococcal polysaccharide capsule is composed of roughly 90% glucuronoxylomannan (GXM) and 10% glucuronoxylomannogalactan (GXMGal, also called GalXM). GXM is a repeating polymer that consists mostly of a linear α-(1-3)-mannan substituted with β-(1, 2)-glucuronic acid and β-(1-4)-xylose (Fig. 1). GXMGal consists of an α-(1-6)-galactan backbone with galactomannan side chains that are further substituted with various numbers of glucuronic acid and xylose residues (24–27).

Although both GXM and GXMGal have immunomodulatory roles (reviewed in reference 28), most studies on the effect of cryptococcal capsular polysaccharide have been performed with GXM. However, although GXM has antigenic properties, naive, wild-type GXM administration is not protective in a murine immunization model (29–31). It may be that SGs act as adjuvants, and their accumulation within the capsule may affect the antigenic properties of GXM components, leading to a much stronger induction of the host immune response.

In this paper, we studied strains deleted for genes whose products transport GXM precursors in the ∆*sgl1* background to decipher whether the lack of glucuronic acid, xylose, or mannose in the capsule would affect the protective role inferred by the accumulation of SGs.

## RESULTS

### Generation of *C. neoformans* capsule component mutants in the *∆sgl1* background

Glucuronic acid, xylose, and mannose are the components of the GXM capsule polysaccharide. Activated donors of these monosaccharides are synthesized in the cytosol and then transported into the secretory pathway, through specific transporters which are: Uut1 (UDP-glucuronic acid transporter), Uxt1 and Uxt2 (UDP-xylose transporters), and Gmt1 and Gmt2 (GDP-mannose transporters) (32). The components are linked through various glycosidic bonds and assembled to form the characteristic capsular microfibrils (Fig. 1) (reviewed in reference 33). Some mannose residues in the mannose backbone can also be acetylated by the Cas1 acetyl transferase.

We wondered whether any of these capsular components would be essential for the protective phenotype observed when SGs accumulate. Using a CRISPR-Cas9 system optimized for *C. neoformans* gene editing, we deleted each transporter in the Δ*sgl1* background to generate ∆*uut1/*∆*sgl1*, ∆*uxt1/*∆*sgl1*, ∆*uxt2*/∆*sgl1,* ∆*uxt1/*∆*uxt2/*∆*sgl1*, ∆*gmt1/*∆*sgl1*, ∆*gmt2/*∆*sgl1*, and ∆*cas1/*∆*sgl1* strains (Table S1; Fig. S1 to S5). All double and/or triple mutant strains were viable and grew well in rich medium (e.g., YPD).

**Fig 2:**
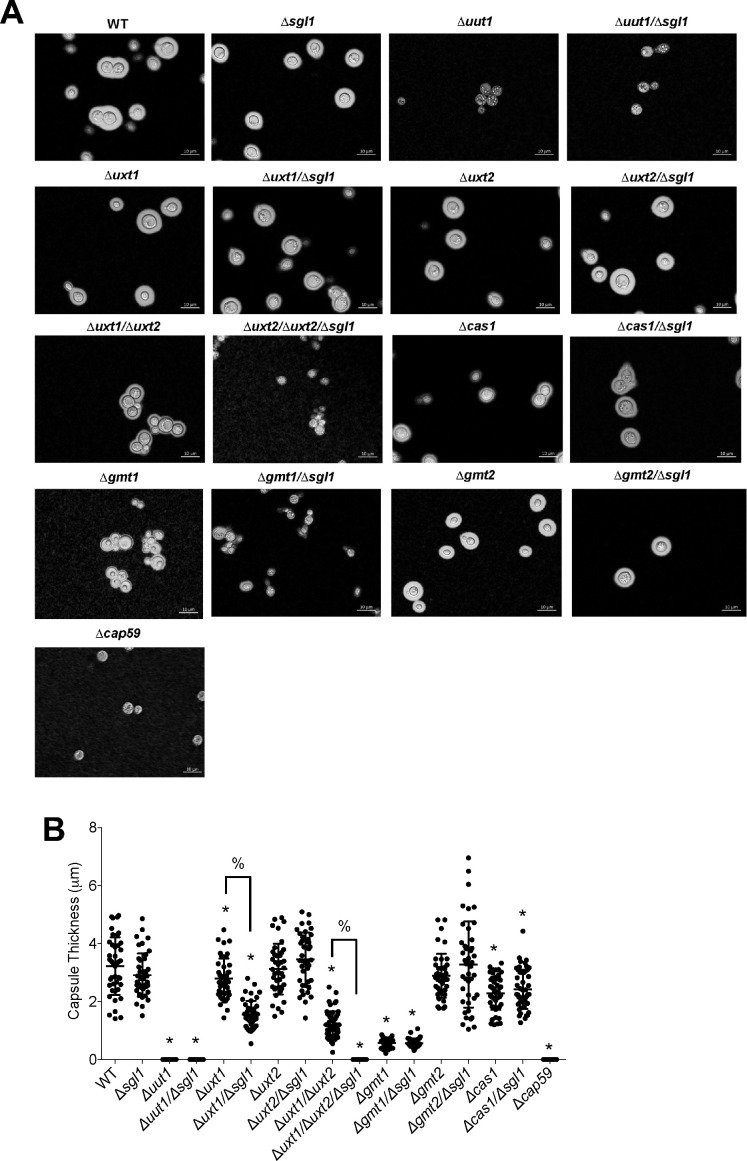
Effect on capsule size when *SGL1* is deleted from strains lacking transporters of GXM precursors. Strains were grown in the capsule-inducing medium at pH 7.4, and capsule measurements were performed by India ink staining. (**A**) Samples were imaged using a Zeiss microscope, and pictures were taken using Zen Pro software. (**B**) Capsule thickness was determined as described in Materials and Methods. *, *P* < 0.05, versus wild type (WT) or *∆sgl1*; %, *P* < 0.05 between the indicated strains. Significance was determined via a one-way ANOVA using Dunnett’s multiple comparison tests for *P* value adjustment.

All single, double, and triple mutants were grown under capsule-inducing conditions, and capsule size was measured using the India ink staining assay. *C. neoformans* wild-type H99 and the ∆*cap59* mutants (totally acapsular) were included as positive and negative controls, respectively. Results showed that, as previously reported (23), deletion of *SGL1* has no effect on capsule size measured by India ink (Fig. 2A and B). Also, capsule size of the ∆*uut1/*∆*sgl1*, ∆*uxt2/*∆*sgl1,* ∆*gmt1/*∆*sgl1*, ∆*gmt2/*∆*sgl1*, or ∆*cas1/*∆*sgl1* double mutant was identical to their respective single mutant in which *SGL1* was not deleted (Fig. 2B). However, the capsule of the ∆*uxt1/*∆*sgl1* double mutant was significantly smaller than the respective ∆*uxt1* or ∆*sgl1* single mutant (Fig. 2B). Similarly, the capsule of the ∆*uxt1/*∆*uxt2/*∆*sgl1* triple mutant was undetectable compared with the capsule of the ∆*uxt1/*∆*uxt2* double mutant (Fig. 2B), suggesting that SG accumulation may interfere with the Uxt2 rather than with the Uxt1 transporter (deletion of Sgl1 does not affect capsule size in the ∆*uxt2* background). Deletion of Uut1 and Gmt1 transporters was already described to dramatically reduce capsule size to a level like the *∆cap59* mutant (34, 35), and our data corroborate those observations.

### Transport of GXM components affects SG localization

In previous studies, we found that deletion of *SGL1* causes an accumulation of SGs in cells, in medium, and, particularly, in the GXM. In fact, the GXM isolated from ∆*sgl1* strain is highly viscous, due to the presence of SGs (23). If the strain does not make GXM, such as the ∆*cap59/*∆*sgl1* mutant, SGs are mainly localized in the cell and in the medium, but not with the GXM extracted fraction (23). Thus, we investigated whether the deletion of each of the nucleotide-sugar transporters would affect SG localization.

**Fig 3:**
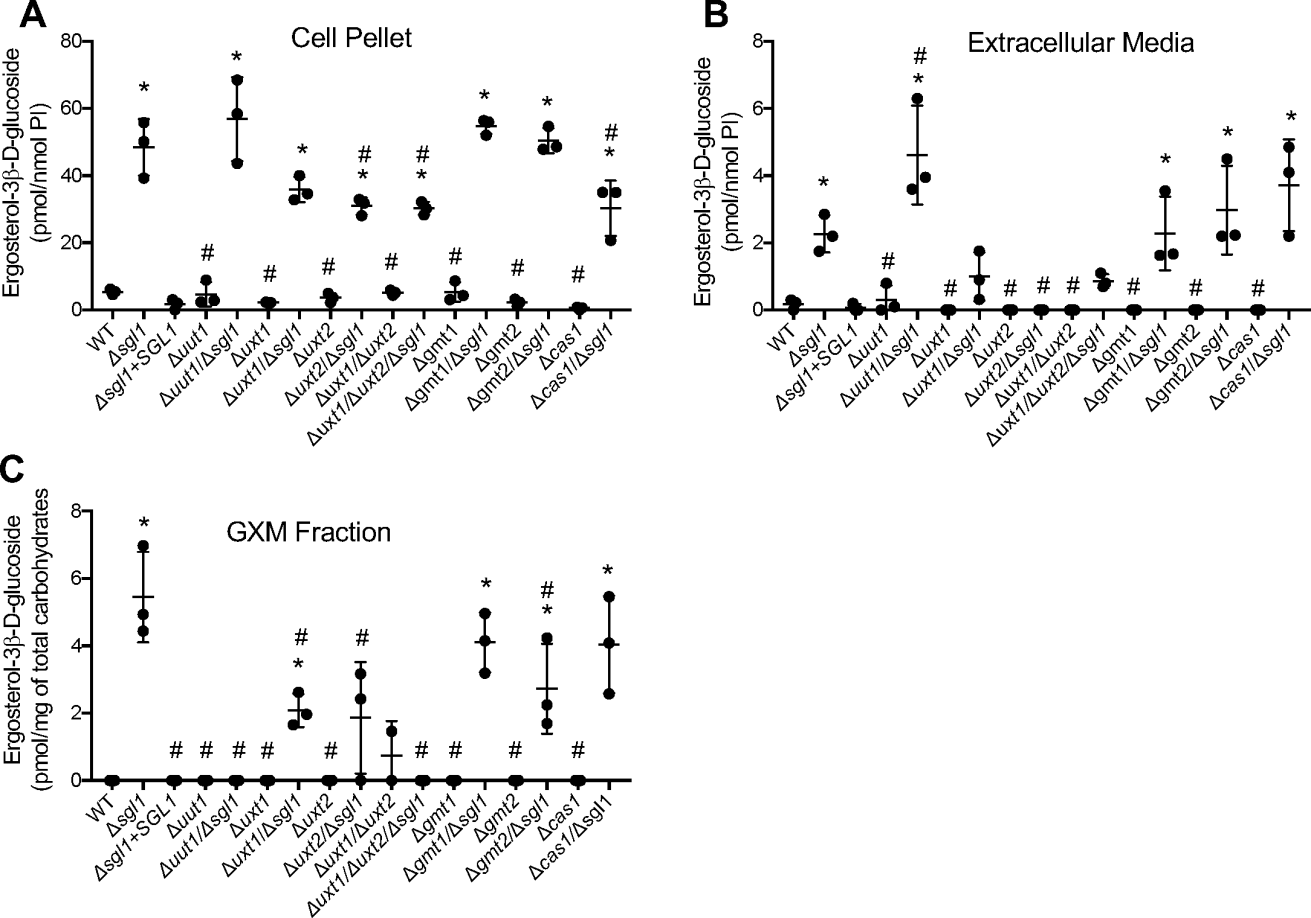
Transport of GXM components affects SG localization. Ergosterol-3β-D-glucoside (the main component of SG), extracted from cell pellet (**A**), extracellular medium (**B**), and GXM fraction (**C**) was analyzed by liquid chromatography mass spectrometry (LC-MS). Uut1, Uxt1, Uxt2, and Gmt2 are required for SGs to be associated with the GXM fraction. *, *P* < 0.05, versus wild-type WT) strain; ^#^, *P* < 0.05 versus *∆sgl1* strain. Significance was determined via one-way ANOVA using Dunnett’s multiple comparison tests for *P* value adjustment.

Using liquid chromatography mass spectrometry (LC-MS), we measured ergosterol-3β-D-glucoside, which is the most abundant sterylglucoside component of SGs (19), in cell pellets, spent growth medium, and in GXM fractions isolated from our mutant strains (Fig. 3). Lack of Uut1 alone did not affect the number of SGs in the cell pellet (Fig. 3A), medium (Fig. 3B), or GXM (Fig. 3C); in all cases, they remained at the low baseline WT levels. Similarly, adding this deletion to ∆*sgl1* cells did not alter SG accumulation in the pellet or medium fractions. However, it strikingly eliminated SG accumulation in GXM (∆*uut1*/∆*sgl1*, Fig. 3C). Deleting *UXT1* or *UXT2* along with *SGL1* (∆*uxt1*/∆*sgl1* or ∆*uxt2*/∆*sgl1*) diminished the level of SGs in the cell pellet and drastically reduced SGs in the GXM fraction and in the medium, compared with the ∆*sgl1* (Fig. 3). Deletion of Gmt1 or Gmt2 transporters in the ∆*sgl1* background did not have a major effect on SG amounts in cell pellet or media, compared with ∆*sgl1* alone. However, we observed a significant decrease of SGs in the GXM fraction of the ∆*gmt2*/∆*sgl1* compared with the ∆*sgl1* single mutant (Fig. 3). A slight reduction of cell pellet-associated SGs was also observed in the ∆*cas1*/∆*sgl1* mutant, but there were no differences in the media or GXM fraction (Fig. 3).

Overall, our results suggest that the transport of UDP-glucuronic acid by Uut1 and UDP-xylose by Uxt1 or Uxt2 into the secretory pathway, where GXM synthesis occurs, is required for SGs to be associated with the GXM fraction when they accumulate. The transport of GDP-mannose by Gmt2 into the secretory pathway is also important for SGs to fully accumulate in the GXM fraction.

### Lack of nucleotide transporters does not alter the avirulence of ∆*sgl1*

To examine the effect of the deletion of nucleotide sugar transporters on virulence when SGs accumulate, we characterized the virulence phenotype of our mutants. Figure S6 illustrates the growth phenotype at 37°C using a physiological medium (DMEM) at pH 4.0 and 7.4. The ∆*sgl1* mutant grows normally in both pH, whereas the ∆*uut1* and the ∆*uut1/*∆*sgl1* show growth arrest during the first 3 days at pH 4.0 and complete arrest at pH 7.4 (Fig. S6A and B). This growth defect is ascribed to the lack of Uut1, as previously described (34). Deletion of GDP-mannose transporters has no impact on growth at pH 4.0 (Fig. S6C), whereas at pH 7.4, we observed growth arrest of ∆*gmt1* and ∆*gmt1/*∆*sgl1* strains (Fig. S6D), again due to the lack of Gmt1 (35). Of interest, the growth defect of ∆*gmt2/*∆*sgl1* double mutant at pH 7.4 was not detected in the ∆*gmt2* single mutant (Fig. S6D) (35). This growth defect persists for 72 h, and the ∆*gmt2/*∆*sgl1* seems to recover growth after 96 hours of incubation. Deletion of the xylose transporters in the ∆*sgl1* background only affects growth when both are deleted (Fig. S6E and F), most likely due to the lack of Uxt1 and Uxt2 instead of SGs accumulation, as previously described (36).

Next, we evaluated the resistance of our mutants to different stress agents, such as potassium chloride, sodium chloride, SDS, Congo Red, calcofluor white, sodium nitrite, and hydrogen peroxide. We did not find any differences among the WT, ∆*sgl1,* or nucleotide-sugar transporter mutants, alone or in combination (Fig. S7). We also measured the extracellular phospholipase activity, considered an important virulence factor (37) and essential for cryptococcal survival in the central nervous system (38). We observed that ∆*uut1* and ∆*uut1/*∆*sgl1* strains had no phospholipase activity, whereas the ∆*uxt1/*∆*sgl1* double mutant had higher phospholipase activity compared with the ∆*uxt1* or ∆*sgl1* single mutant (Fig. S8). Finally, we measured urease activity and melanin production in our mutant strains (Fig. S9). Urease activity is involved in the regulation of many metabolic pathways in *C. neoformans,* including melanin production (39), whereas melanin not only regulates cryptococcal growth (40) but, importantly, affects lung inflammatory responses during cryptococcal infection (reviewed in references 41–43). We found that the ∆*uut1* and ∆*uut1/*∆*sgl1* were the only mutants showing low urease activity compared to WT or ∆*sgl1* (Fig. S9A and B); as with the low phospholipase activity of these mutants, this is most likely ascribed to the lack of Uut1. We found no difference in melanin production in all tested strains (Fig. S9C). Overall, our results showed that most *in vitro* virulence-associated phenotypes of nucleotide sugar mutants are neither diminished nor exacerbated by the deletion of Sgl1. This suggests that the accumulation of SGs does not significantly affect these phenotypes.

**Fig 4:**
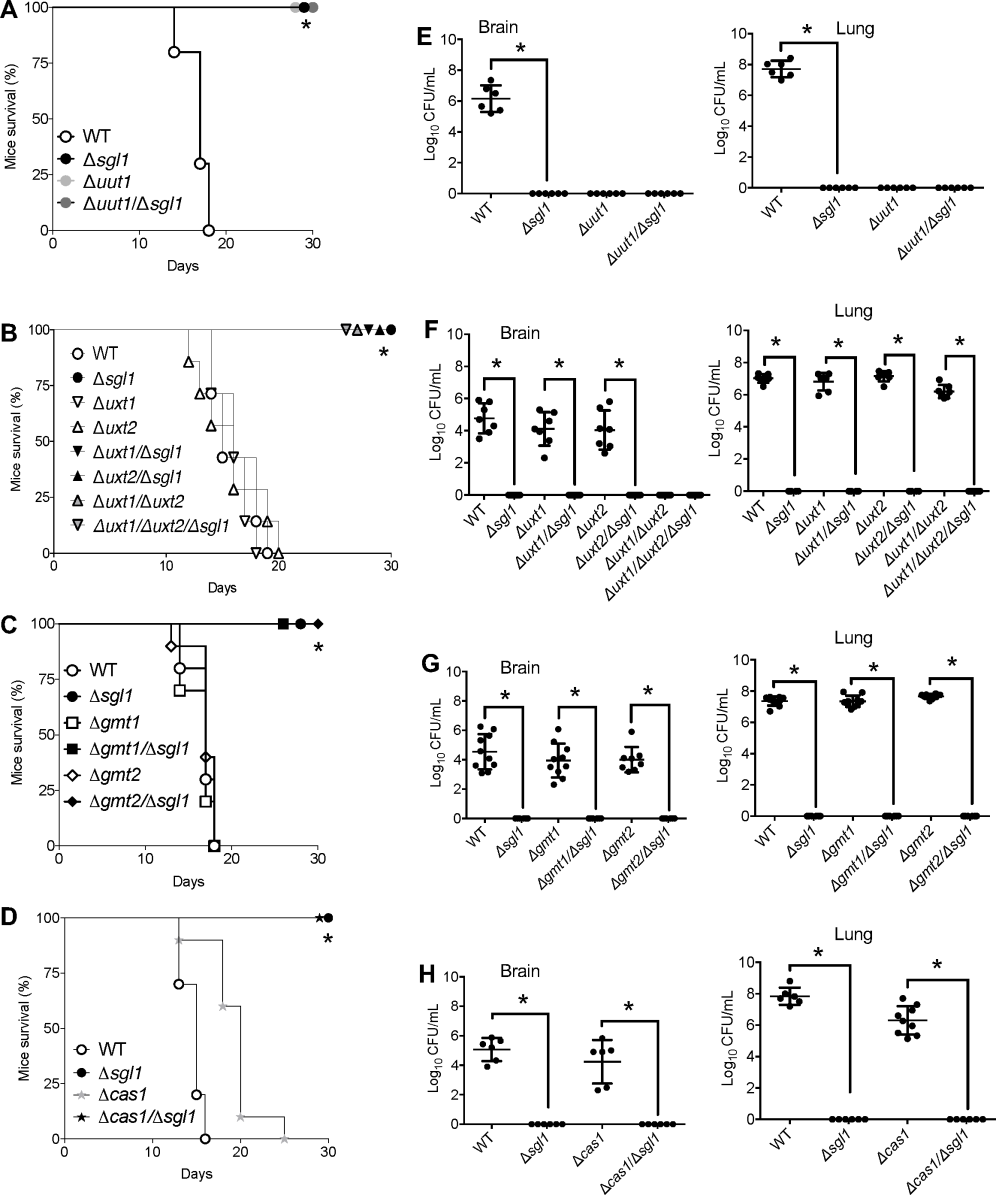
Deletion of the *SGL1* gene renders the respective double mutant avirulent. Mice survival (**A-D**) and tissue burden culture (TBC) by colony forming unit (CFU) analysis (**E-H**) of CBA/J mice infected intranasally with 5 × 10^5^ *C. neoformans* cells. The ∆*uut1/*∆*sgl1,* ∆*uxt1/*∆*sgl1,* ∆*uxt2/*∆*sgl1,* ∆*gmt1/*∆*sgl1,* ∆*gmt2/*∆*sgl1,* and ∆*cas1/*∆*sgl1* double mutants are avirulent, like the ∆*sgl1* mutant. Average survival is as follows: (A) WT 16.7 ± 1.49 days; (**B**) WT 16 ± 2 days, ∆*uxt1* 16 ± 1.56 days, ∆*uxt2* 15.2 ± 2.74 days; (**C**) WT 16.9 ± 1.1 days, ∆*gmt1* 17 ± 1.82 days, ∆*gmt2* 17.9 ± 2.02 days; (**D**) WT 14.6 ± 1.17 days, ∆*cas1* 19.2 ± 2.97 days. In A, B, C, and D *, *P* < 0.05 versus wild type (WT) strain. Statistical analysis performed by rank (Mantel-Cox) test. Values are expressed as mean ± standard error. In E, F, G, and H, *, *P* < 0.05 between the indicated strains. Values are expressed as mean ± standard error. Statistical analysis was conducted using one-way analysis of variance (ANOVA), followed by Tukey’s post hoc test for multiple comparisons.

To determine if the avirulence of *∆sgl1* in mice would be impacted when nucleotide sugar transporters were deleted, we infected mice and evaluated their survival over 30 days of observation (Fig. 4). Mice were male and female CBA/J 3- to 4-week-old and were intranasally infected with 5 × 10^5^ cryptococcal cells. We found that except for the ∆*uut1* mutant, which is avirulent (34), deletion of *SGL1* eliminated the virulence phenotype of their respective single nucleotide sugar transporter mutant (Fig. 4A through D). In fact, the single mutants of ∆*uxt1*, ∆*uxt2*, ∆*gmt1*, ∆*gmt2,* and, to a lesser extent, ∆*cas1* are similarly virulent to the WT *C. neoformans* strain. Deletion of the *SGL1* gene renders the respective double mutant (∆*uxt1/*∆*sgl1,* ∆*uxt2/*∆*sgl1,* ∆*gmt1/*∆*sgl1,* ∆*gmt2/*∆*sgl1,* and ∆*cas/*∆*sgl1*) totally avirulent, like the ∆*sgl1* mutant.

Most interestingly, *SGL1* deletion, and thus SG accumulation, not only renders strains avirulent but also removes their ability to survive in the lung (Fig. 4E through H). In fact, at the end-point (30 days post-infection), no colony-forming units (CFUs) were recovered from the lung of any mouse that was infected with the double or triple mutant in which Sgl1 was deleted. Furthermore, none of the mutants in which *SGL1* was deleted was recovered from the brain, suggesting that accumulation of SGs abrogated the ability of the fungus to reach or to survive in the brain tissue, where it normally caused the characteristic meningoencephalitis (Fig. 4E through H).

**Fig 5:**
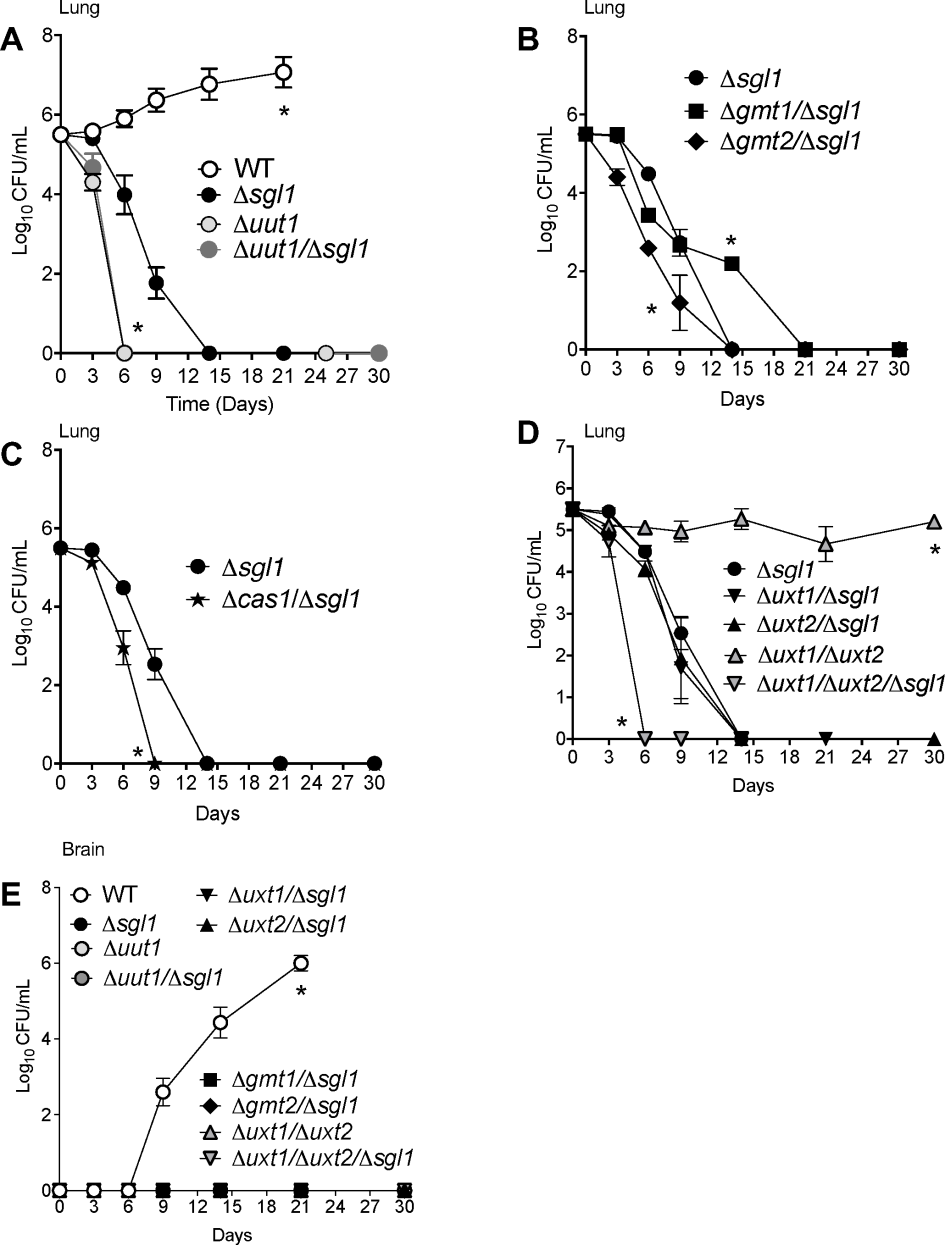
Deletion of *SGL1* abrogates cryptococcal virulence. CBA/J mice were intranasally infected with 5 × 10^5^ *C. neoformans* cells, and at the indicated days, three mice per strain were euthanized, and their organs were collected and processed for tissue burden analysis using CFUs. CFU analysis was performed at 3, 6, 9, 14, 21, and 30 days post-infection for lungs (**A-D**) and brain (**E**). Values are expressed as mean ± standard error. *, *P* < 0.05 between the indicated strain versus the WT (in A) or versus *∆sgl1* (in B-D). In E, *, *P* < 0.05 between the WT versus any mutant. Statistical analysis was conducted using one-way analysis of variance (ANOVA), followed by Tukey’s post hoc test for multiple comparisons.

The CFU analysis illustrated in Fig. 4 was done either at the time of death (for the WT, ∆*uxt1,* ∆*uxt2,* ∆*gmt1,* ∆*gmt2,* and ∆*cas1*) or at the 30-day end-point when all mice were euthanized (for ∆*sgl1,* ∆*uut1,* ∆*sgl1/*∆*uut1,* ∆*uxt1/*∆*uxt2,* ∆*uxt1/*∆*uxt2/*∆*sgl1,* ∆*gmt1/*∆*sgl1,* ∆*gmt2/*∆*sgl1,* and ∆*cas1/*∆*sgl1*). Thus, to better understand the dynamics of the cell survival of the cryptococcal avirulent mutants, we performed a time course analysis of the CFU of lung and brain in early (3, 6, and 14 days) and late (21 and 30 days) stages of infection. To do this, CBA/J mice were intranasally infected with 5 × 10^5^ *C. neoformans* cells, and at the indicated days, three mice per strain were euthanized, and organs were collected and processed for CFU analysis. We found that deletion of *SGL1* results in rapid elimination of the fungal mutant from the lung by day 14 (Fig. 5A through D), with the ∆*gmt1/*∆*sgl1* being eliminated by day 21 (Fig. 5B). Note the persistence of the ∆*uxt1/*∆*uxt2* cells in the lung during the entire observation period (30 days) and the extremely rapid elimination (by day 6) of the mutant when *SGL1* deletion is added (∆*uxt1/*∆*uxt2*/∆*sgl1*) (Fig. 5D). Analysis of brain tissue revealed that *C. neoformans* WT cells are observed by day 9, whereas none of the mutant cells in which *SGL1* was deleted were present in the brain at any time point (Fig. 5E). Taken together, our results indicate that deletion of *SGL1* abrogates cryptococcal virulence by impairing fungal survival in the lung environment, not only when introduced in the wild-type strain but also when introduced in various mutant transporters involved in capsular formation.

### Deletion of transporters affects vaccine protection when SGs accumulate

We previously reported that deletion of *SGL1* and accumulation of SGs is necessary but not sufficient to induce protection against a secondary challenge: GXM must be present because vaccination with a strain in which *SGL1* is deleted but without GXM (∆*cap59/*∆*sgl1*) does not confer any protection against a secondary challenge.

To test if any of our mutants would confer protection, we first administered 5 × 10^5^ cells of our avirulent strains intranasally to CBA/J mice. After 30 days, we similarly infected with the virulent *C. neoformans* WT strain H99 and then observed mouse survival for an additional 60 days. During the 60-day period, we assessed lung and brain burden at the time of death of each mouse that succumbed to the infection. The remaining mice that survived the challenge were euthanized at the end of the study (60 days), and CFUs of lungs and brains were measured at that time (end point CFU). Samples for CFU from vaccinated mice were plated on YPD with or without either 125 µg/mL of nourseothricin or 300 µg/mL of hygromycin, according to the mutant administered, to make sure that the fungus recovered from lungs or brains was the WT strain and not the mutants. The *∆uut1/∆sgl1, ∆uxt1/∆sgl1, ∆uxt2/∆sgl1, ∆uxt1/∆uxt2∆sgl1, ∆gmt1/∆sgl1, ∆gmt2/∆sgl1,* and *∆cas1/∆sgl1* mutants contain the *HYG^R^* and the *NAT1* genes and are thus resistant to both hygromycin and nourseothricin, whereas the *∆sgl1* contains only the *NAT1* gene and is thus resistant to only nourseothricin.

**Fig 6:**
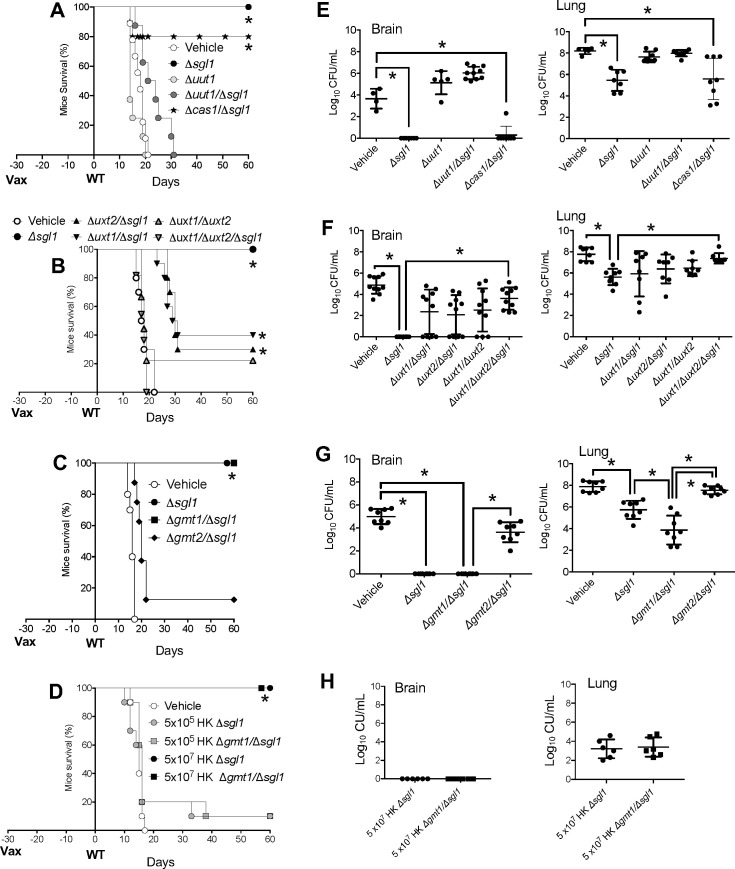
Deletion of GXM transporters affects vaccine protection when SGs accumulate. Survival (**A-D**) and CFU analysis (**E-H**) of CBA/J mice vaccinated (Vax) with 5 × 10^5^ cells of avirulent *C. neoformans* mutant(s) and challenged after 30 days with 5 × 10^5^ cells of wild-type (WT) *C. neoformans*. Only ∆*sgl1* and ∆*gmt1/*∆*sgl1* administration provided full protection. Average survival is as follows: (A) vehicle 18 ± 2.44 days, ∆*uut1* 15.8 ± 2.69 days, and ∆*uut1/*∆*sgl1* 23 ± 5.41 days; (**B**) vehicle 18.2 ± 2.82 days, and ∆*uxt1/*∆*uxt2/*∆*sgl1* 17.5 ± 1.5 days; (**C**) vehicle 17.6 ± 1.64 days; (**D**) vehicle 17.1 ± 1.19 days. In A, B, C, and D *, *P* < 0.05 versus wild-type (WT) strain. Statistical analysis performed by rank (Mantel-Cox) test. Values are expressed as mean ± standard error. In E, F, G, and H, *, *P* < 0.05 between the indicated strains. Values are expressed as mean ± standard error. Statistical analysis was conducted using one-way analysis of variance (ANOVA), followed by Tukey’s post hoc test for multiple comparisons.

We found that strains lacking Uut1, Uxt1, Uxt2, or Gmt2 in the *∆sgl1* background significantly reduced or totally abrogated protection (Fig. 6A through C). This lack of protection is reflected by the numerous WT cells present in the lung and in the brain at the time of death (Fig. 6E through G). In contrast, deletion of *CAS1*, and particularly *GMT1*, did not significantly change the protective effect of *SGL1* deletion (Fig. 6A and C). In fact, vaccination with the *∆cas1/∆sgl1* conferred ~80% protection (Fig. 6A), whereas vaccination with the *∆gmt1/∆sgl1* conferred 100% protection (Fig. 6C), identical to the 100% protection conferred by the *∆sgl1* single mutant. End-point lung and brain CFUs (at 30 days) show a similar cell number between *∆cas1/∆sgl1* and *∆sgl1* vaccinated mice (Fig. 6E). However, it is interesting that the lung CFUs of the *∆gmt1/∆sgl1*-vaccinated mice were significantly lower than the *∆sgl1-*vaccinated mice (Fig. 6G). It is intriguing that the deletion of *GMT2* abrogates, whereas the deletion of *GMT1* retains protection in the *∆sgl1* background, consistent with our prior conclusions that these two GDP-mannose transporters have distinct biological roles (32).

In previous studies, we showed that ∆*sgl1* cells are fully protective not only when administered live but also when administered heat-killed (44). Thus, because of the striking protective phenotype of the *∆gmt1/∆sgl1* vaccination, we performed an additional vaccination experiment in which we intranasally administered *∆gmt1/∆sgl1* as heat-killed cells. As a positive control, we used *∆sgl1* heat-killed cells. We found that similar to the heat-killed *∆sgl1* cells, the *∆gmt1/∆sgl1* heat-killed cells conferred a dose-dependent protection, with 5 × 10^7^ cells being necessary to fully protect the mice (Fig. 6D). The lung end-point CFU recovered from the survived mice vaccinated with either *∆gmt1/∆sgl1* or *∆sgl1* was very similar (Fig. 6H). Of interest, the administration of the highest dose of heat-killed cells (5 × 10^7^) provided better lung clearance compared with the administration of 5 × 10^5^ live cells (compare lung CFUs in Fig. 6H with lung CFUs in Fig. 6G), validating previous results (21, 44). Overall, our data show that the availability of precursors for glucuronic acid and xylose, together with SG accumulation, is essential for host protection. Because *∆gmt1/∆sgl1*, but not *∆gmt2/∆sgl1*, administration is protective, our results also show that GDP-mannose specifically transported by Gmt2 but not by Gmt1 is important for host protection.

### γδ T cell response is affected by the deletion of nucleotide sugar transporters in the condition of SG accumulation

**Fig 7:**
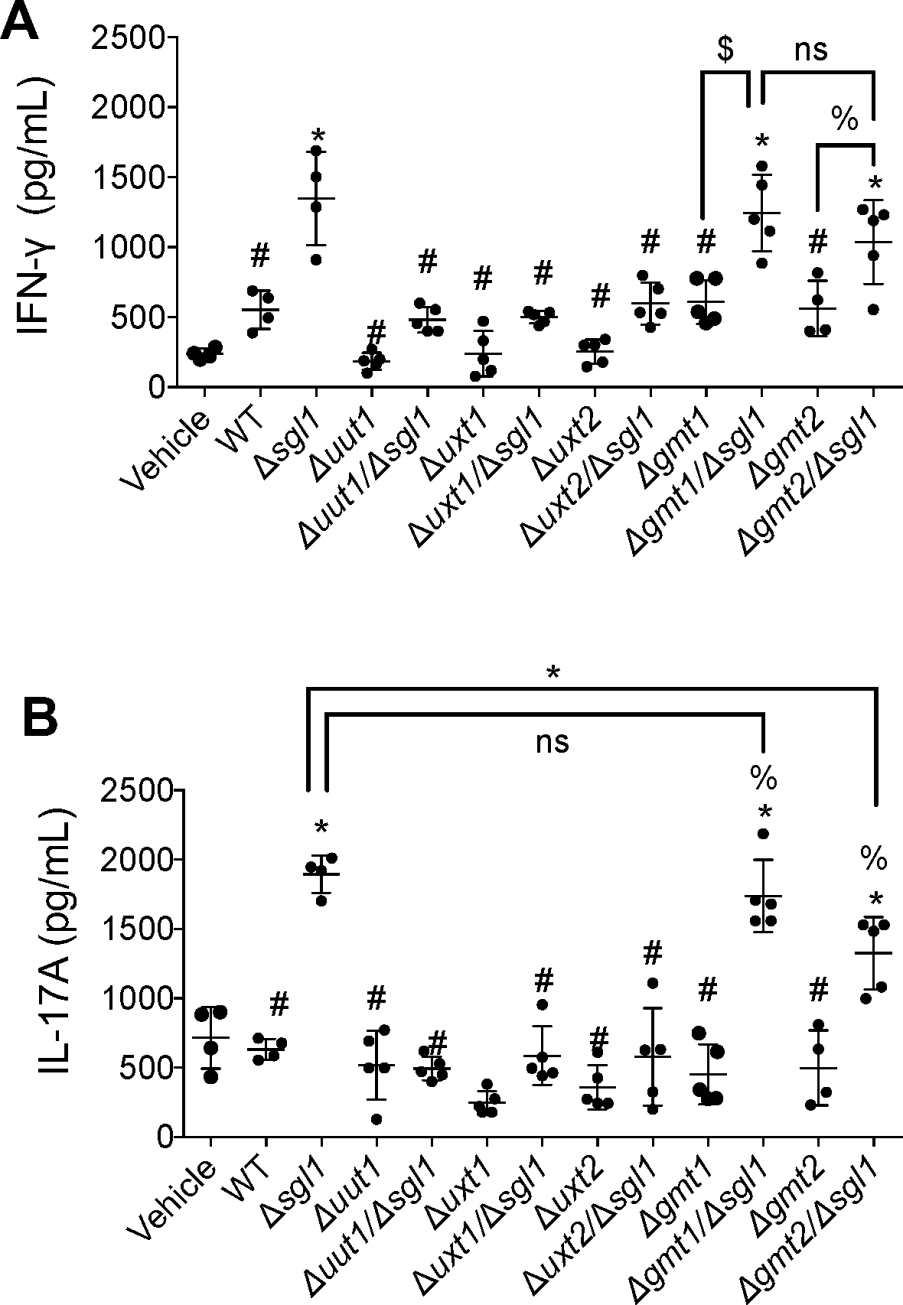
γδ T cell response is affected by the deletion of nucleotide sugar transporter. Evaluation of inflammatory interleukin secretion by γδ T cells. IFNγ (**A**) and IL-17A (**B**) were measured after a 5-day exposure of γδ T cells to capsule mutants. Glucuronic acid, xylose, and mannose organization on the cryptococcal capsule is important to stimulate a protective cytokine response by γδ T cells in the presence of SGs. *P < 0.05 versus the WT strain; ^#^, *P* < 0.05 versus the *∆sgl1* strain; $, *P* < 0.05 versus the indicated groups; % *P* < 0.05 versus WT; ns, not significant. Values are expressed as mean ± standard error. Statistical analysis was conducted using one-way analysis of variance (ANOVA), followed by Tukey’s post hoc test for multiple comparisons.

In previous studies, we provided a comprehensive analysis of the immunity required for ∆*sgl1* to induce a protective effect (21). Notably, production of IFNγ and IL-17A by γδ T cells is necessary (21). We therefore examined the production of these cytokines after exposing γδ T cells to our capsular mutants (Fig. 7A and B). We found that deletion of *UUT1* or *UXT1* in the *∆sgl1* background totally abrogated the production of IFNγ (Fig. 7A) and IL-17A (Fig. 7B) by γδ T cells stimulated by the accumulation of SGs. Interestingly, *∆gmt1/∆sgl1* and *∆gmt2/∆sgl1* double mutants stimulate IFNγ production like the *∆sgl1* single mutant (Fig. 7A). Also, IL-17A stimulation by *∆gmt2/∆sgl1* mutant is significantly lower compared with the IL-17A stimulation by *∆sgl1* alone and lower compared with IL-17A stimulation by *∆gmt1/∆sgl1* mutant, although this difference was not statistically significant. These results suggest that glucuronic acid, xylose, and mannose organization on the cryptococcal capsule is important to stimulate a protective cytokine response by γδ T cells in the presence of SGs.

### Effect of nucleotide sugar transporter deletions on the binding of anti-GXM antibody and on phagocytosis by macrophages

**Fig 8:**
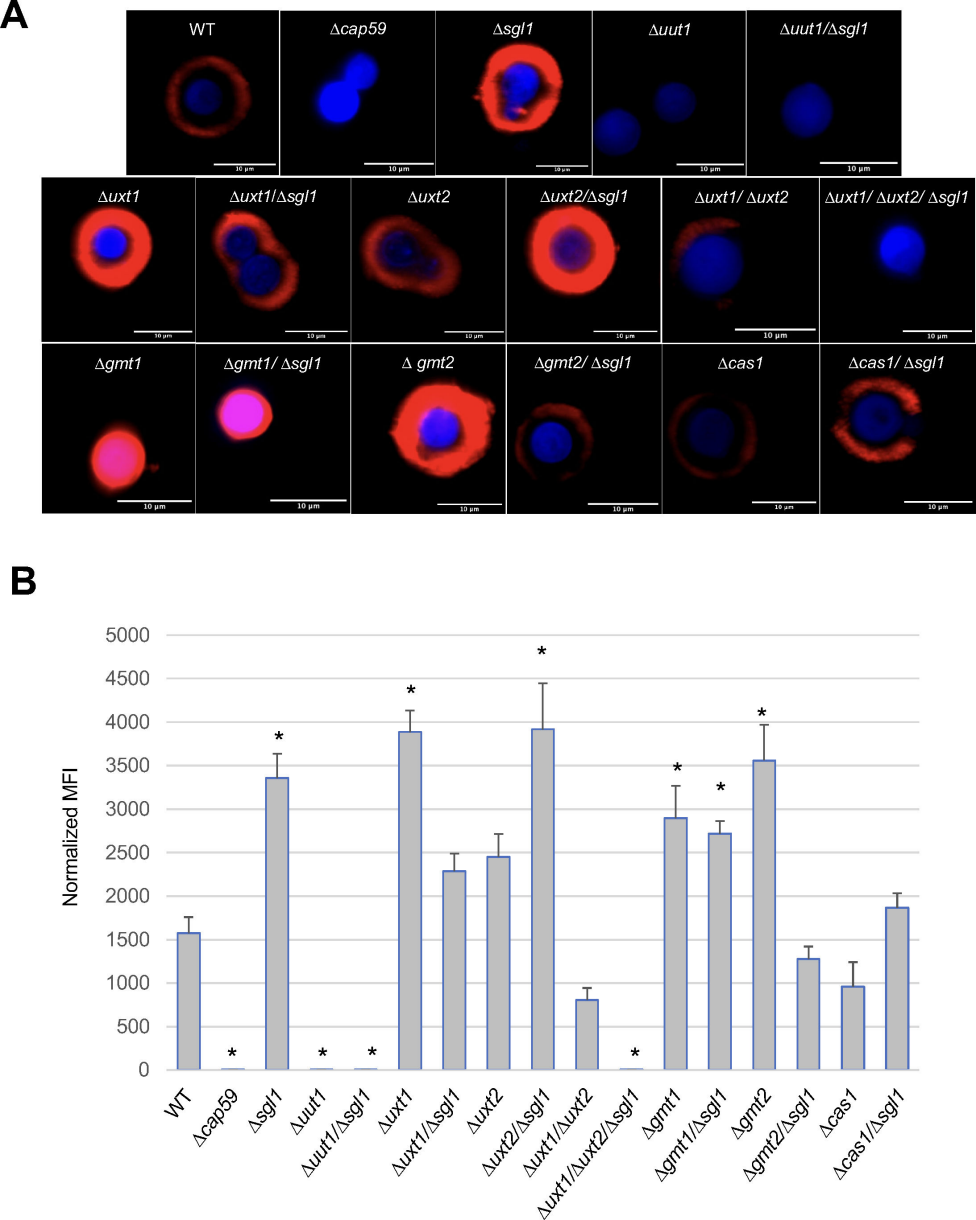
Analysis of mAb 18B7 staining of *C. neoformans* capsular surface. *C. neoformans* was grown in capsule-inducing conditions and stained with anti-GXM antibody 18B7 followed by a fluorescent secondary antibody conjugated to Alexa Fluor 568 (red). Cell wall chitin was stained using calcofluor white (in blue). (**A**) Images were taken at 100× magnification using a Zeiss Axio observer microscope (Thornwood, NY). (**B**) Fluorescence intensity was quantified, and the average fluorescence was measured using ImageJ/Fiji software. Mean fluorescence intensity values (MFI) derived from at least 50 cells were normalized by subtracting the MFI of the acapsular strain *∆cap59*, which was the control for the capsular staining background. Results show that SG accumulation affects the binding of 18B7 to GXM when the level of glucuronic acid, xylose, or mannose is altered. *, *P* < 0.05 versus WT. Values are expressed as mean ± standard error. Statistical analysis was conducted using one-way ANOVA, followed by Tukey’s post hoc test for multiple comparisons.

The mouse monoclonal antibody 18B7 binds specifically to cryptococcal GXM. It binds to GXM of various cryptococcal species with different intensities. Thus, we wondered whether the accumulation of SGs and/or the concomitant deletion of various nucleotide sugar transporters would affect the binding of 18B7 to GXM. We found that 18B7 did not bind to ∆*uut1*, ∆*uut1*/∆*sgl1,* ∆*uxt1*/∆*uxt2*/∆*sgl1,* or ∆*cap59* (Fig. 8), consistent with the observation that these strains do not produce any capsule (Fig. 2) (34). Notably, the ∆*sgl1* single mutant exhibited high levels of 18B7 binding to GXM compared with WT (Fig. 8A and B). In general, mutants with capsules similar to WT, such as ∆*uxt1,* ∆*uxt2,* ∆*uxt1/*∆*sgl1,* ∆*uxt2/*∆*sgl1,* and ∆*gmt2*, also showed clear 18B7 binding. However, some mutants with very small capsules, such as ∆*gmt1* and ∆*gmt1*/∆*sgl1*, still showed strong 18B7 binding, although the staining was localized differently. In these mutants, 18B7 seems to mainly bind the cell wall, as the GXM staining overlaps with calcofluor white (CFW) staining of that structure. Other mutants, such as ∆*gmt2*/∆*sgl1*, showed a substantial reduction in 18B7 binding compared with ∆*gmt2* or ∆*sgl1* (Fig. 8). It is interesting to note that in some double mutants, accumulation of SGs causes stronger deposition of 18B7 (compare ∆*uxt2* with ∆*uxt2/*∆*sgl1* and ∆*cas1* with ∆*cas1/*∆*sgl1*), whereas in other mutants, accumulation of SGs causes a decrease in 18B7 deposition (compare ∆*uxt1* with ∆*uxt1/*∆*sgl1* and ∆*gmt2* with ∆*gmt2/*∆*sgl1*). Taken together, these results suggest that the interaction of mAb 18B7 with the cryptococcal surface is affected by a complex combination of GXM composition and SG accumulation.

The differential binding of 18B7 with various strains suggests changes in the nature of their surfaces. To determine whether this would affect phagocytosis, we examined the uptake of our mutant strains by macrophages after opsonization with mAb 18B7 alone (no complement). The *∆sgl1* mutant and the WT strain are similarly phagocytosed by macrophages, as previously reported (19). In contrast, we observed a remarkable decrease in the phagocytic index of all capsular mutants compared with WT, regardless of SG accumulation, although strains that lacked only one Uxt showed somewhat less impairment (Fig. S10). There was no correlation between 18B7-GXM intensity (Fig. 8) and phagocytic index (Fig. S10). These findings suggest that SG accumulation in the capsule affects the interaction of 18B7 with GXM when the level of glucuronic acid, xylose, or mannose is altered, without significantly compromising fungal opsonization.

### Accumulation of SGs may affect GXM size as analyzed by dynamic light scattering

**Fig 9:**
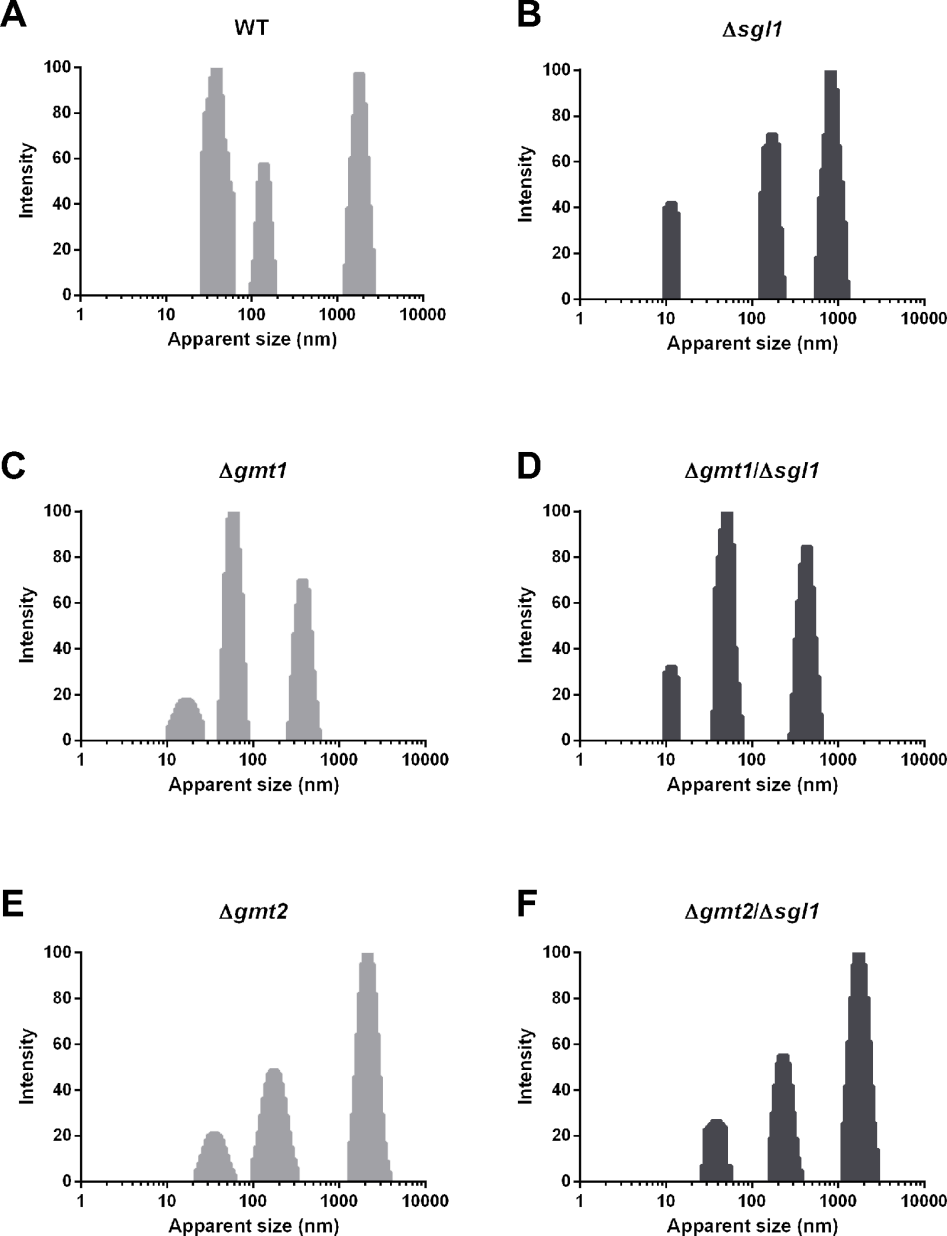
Size distribution of capsular GXM by DLS. The size distribution of GXM from *C. neoformans* wild-type (WT), *∆sgl1*, and nucleotide-sugar transporter mutants *∆gmt1, ∆gmt1/∆sgl1, ∆gmt2,* and *∆gmt2/∆sgl1* was analyzed using DLS. The x-axis represents the measured particle size distribution, whereas the y-axis corresponds to the intensity-weighted size percentages. Results suggest that the accumulation of SGs may affect the peak shape without causing a major difference in GXM size. The results shown are representative of at least three separate experiments.

Dynamic light scattering (DLS) is a technique that analyzes the size and characteristics of the GXM (45). It measures the fluctuation in light scattered by particles in solution, which is related to their size and movement, allowing calculations of hydrodynamic radius. Thus, we subjected GXM from our mutants to DLS analysis to determine their average size, size distribution, and potential aggregation patterns. We found that deletion of *SGL1* (*∆sgl1*) decreases the size of most GXM fibers compared with WT (Fig. 9A and B). Very interestingly, the deletion of *GMT1* (*∆gmt1*) reduced fiber size even more strongly, although this reduction was not changed by the addition of *SGL1* deletion (*∆gmt1/∆sgl1*) (compare Fig. 9C and D). Similarly, there was no major change in fiber size between the *∆gmt2* and the *∆gmt2/∆sgl1* mutant (Fig. 9E and F).

The GXM fiber sizes and distribution between the *∆sgl1* (Fig. 9B) and the *∆gmt1/∆sgl1* (Fig. 9D) mutants have some similarities, which may correlate with the full protective phenotype observed with these mutants (Fig. 6C and D) but not with the *∆gmt2/∆sgl1* mutant (Fig. 6C) that has a quite distinct profile (Fig. 9F). In addition, *SGL1* deletion in the *∆cas1* background (*∆cas1/∆sgl1*, which shows ~80% protection) also reduces the overall size of GXM fibers (Fig. S11A and B). The *∆uxt1* and *∆*uxt2 strains have GXM profiles that are quite different from the others, although notably they show no overall reduction in GXM fiber size, even when *SGL1* deletion is added (Fig. S11C through F). These results suggest that the accumulation of SGs in the capsule may change the peak shape without causing a major difference in GXM size. DLS analysis was not performed in *∆cap59, ∆uut1, ∆uut1/∆sgl1, ∆uxt1/∆uxt2,* and *∆uxt1/∆uxt2/∆sgl1* because no GXM was isolated from these mutants.

### Accumulation of SGs alters GXM architecture analyzed by ^1^H-nuclear magnetic resonance (1D-NMR) spectroscopy

**Fig 10:**
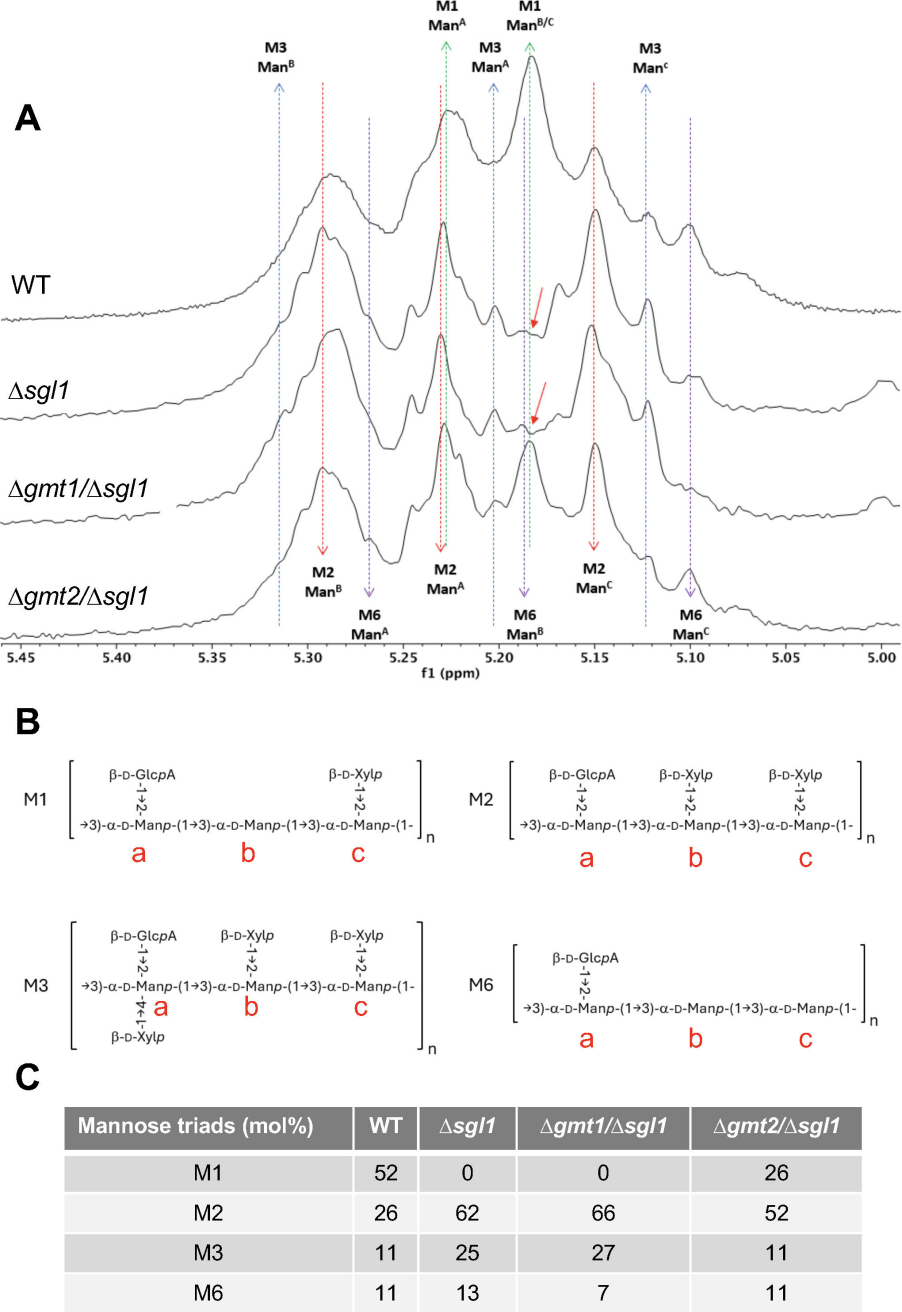
Accumulation of SGs alters GXM architecture by 1D-NMR spectra analysis. (**A**) Comparison ofα-mannose anomeric region of 1D-NMR spectra of the GXM isolated from *C. neoformans* WT, *∆sgl1, ∆gmt1/∆sgl1,* and *∆gmt2/∆sgl1*. Red arrows indicate the absence of M1 in *∆sgl1* and *∆gmt1/∆sgl1* strains. The spectra indicate that the *∆sgl1* and the *∆gmt1/∆sgl1* mutant lack the M1 mannose triad, which is the major triad in the WT strain. (**B**) Composition of the four types of mannose triads. (**C**) Measurement of the mol% of the different mannose triad elements estimated by 1D-NMR analysis.

To gain further structural insights into the small-size fibers observed in the *∆sgl1* and in the *∆gmt1/∆sgl1* mutant, we subjected their GXM to 1D-NMR. This method may be used to assess the various mannose triads present in GXM. As controls, we used the GXM isolated from the WT strain and from the *∆gmt2/∆sgl1* mutant (Fig. 10). By comparing the mannose anomeric proton region of the 1D-NMR spectra, we found a similar pattern between the *∆sgl1* and *∆gmt1/∆sgl1* (Fig. 10A), whereas the *∆gmt2/∆sgl1* pattern had a spectrum similar to that of the WT strain (Fig. 10A). These spectra indicate that the *∆sgl1* and the *∆gmt1/∆sgl1* mutant lack the M1 mannose triad, which is the major triad in the WT strain (Fig. 10B and C). In place of this, the *∆sgl1* and the *∆gmt1/∆sgl1* mutant are enriched in the M2 and the M3 mannose triads, compared with the WT or the *∆gmt2/∆sgl1* (Fig. 10C). GXM samples were also analyzed by trimethylsilyl (TMS) derivatization and gas chromatography mass spectrometry (GC-MS) of the O-TMS methyl glycoside derivatives of the monosaccharides. We did not observe a significant alteration in the overall amount of xylose, glucuronic acid, or mannose among the WT, *∆sgl1, ∆gmt1/∆sgl1,* or *∆gmt2/∆sgl1* samples (Fig. S12).

## DISCUSSION

In previous studies, we showed that deletion of *SGL1* leads to a mutant that accumulated SGs and can be successfully used as a live-attenuated vaccine to protect against subsequent cryptococcal infection (19). We also showed that this protection depends on the presence of the cryptococcal capsule (23). In this study, we analyzed the requirement for each of the capsular components (xylose, glucuronic acid, and mannose) in virulence and protective phenotypes when SGs accumulate. We found that the deletion of *SGL1* and consequent accumulation of SGs may alter GXM structure.

The GXM structure is built on a backbone of α−1,3-linked mannose. For every three mannoses, there is one glucuronic acid residue in β−1,2-linkage and between zero and three xylose residues in β−1,2 and β−1,4 linkage (Fig. 10B) (27, 46). The relative proportion of these mannose trimers, which differ in the extent of xylose and 6-O-acetyl modification, can vary (47). These differences, which also define the species of *C. neoformans*, confer distinct virulence phenotypes and antigenic properties (48).

To dissect the key features of GXM that are required for SG accumulation to be protective, we investigated mutants lacking nucleotide sugar transporters, which are essential to provide the precursors for glycosylation reactions like those involved in GXM synthesis. We found that the varying virulence phenotypes of these mutants in mice were all dominated by the lack of Sgl1, such that all the double or triple mutants that we constructed were avirulent. This allowed us to test their efficacy as live-attenuated vaccine strains.

Based on our results presented here and previously, we hypothesize that SGs act as a potent adjuvant for antigens present on the fungal surface, resulting in successful stimulation of protective immunity. This idea is supported by a study in *Aspergillus fumigatus* in which the deletion of SglA (the Sgl1 homolog) strongly accumulated SGs. The *A. fumigatus ∆sgla* mutant is avirulent, and the administration of live or heat-killed *∆sgla* conidia totally protects against secondary aspergillosis. The protective phenotype was ascribed to the adjuvant effect that these fungal glycolipids (SGs) have on fungal surface antigens. In the case of *C. neoformans*, we further hypothesize that the key antigen is GXM because the absence of GXM eliminates the protective effect.

We suspected that significant alteration of GXM, even without its complete absence, might be enough to eliminate the protective effect of *SGL1* deletion. To test this, we examined strains that cannot transport UDP-glucuronic acid (in *∆uut1* cells) or UDP-xylose (in *∆uxt1/∆uxt2* cells), which would eliminate the corresponding GXM side chains. Indeed, these mutations abolished the protection conferred by the deletion of *SGL1*.

We could not test cells that completely lack the ability to transport GDP-mannose because mutants lacking both known transporters have poor viability. Instead, we tested each transporter separately. Interestingly, we observed opposite vaccine phenotypes with *∆gmt1/∆sgl1* (protective) and *∆gmt2/∆sgl1* (non-protective) mutants. This was particularly surprising because the capsule of the *∆gmt1* mutant is generally smaller than the capsule of the *∆gmt2* mutant. However, the capsule size by India ink does not necessarily reflect its structure, composition, or antigenic properties. In fact, 18B7 deposition of the mAB 18B7 on the fungal surface of the *∆gmt1/∆sgl1* was still very strong despite the small capsule. The deposition of 18B7 on the fungal surface was also very strong in the *∆gmt2* mutant but significantly diminished in the *∆gmt2/∆sgl1*, suggesting that the accumulation of SGs in *∆gmt2* may be detrimental for the interaction between 18B7 and GXM.

When we measured the SG content in the cell pellet, media, and GXM fraction, we found that SG content in the GXM fraction of *∆gmt2/∆sgl1* trended lower than the SG content of *∆gmt1/∆sgl1* and was significantly less than the SG content associated with the GXM fraction of *∆sgl1* (Fig. 3). It may be that the unique GXM architecture of the *∆gmt2/∆sgl1* is unfavorable for the association of SGs, which are then released into the media. In fact, there is a trend of more SGs in the media of *∆gmt2/∆sgl1* compared with *∆gmt1/∆sgl1*, thus decreasing the adjuvant effect of SGs when associated with the capsule.

The *∆gmt1/∆sgl1* and *∆gmt2/∆sgl1* double mutants are the only strains, besides the *∆sgl1* mutant itself, that significantly stimulate the production of IFNγ and IL-17A by γδ T cells. This γδ T stimulation seems to be necessary but not sufficient to induce a protective phenotype because vaccination with the *∆gmt2/∆sgl1* does not induce any protection against a secondary infection, despite similar IFNγ stimulation to the other two strains. Of note, stimulation of IL-17A by *∆gmt2/∆sgl1* is lower than that of *∆sgl1* or *∆gmt1/∆sgl1*. There may be a tipping point, where the production of IL-17A in the lung is not enough to provide even partial protection.

Of note, the *∆gmt2/∆sgl1* clearance from the lung is significantly faster than the clearance of *∆sgl1* or *∆gmt1/∆sgl1* (Fig. 5B). This may be due to the poor adaptation of the *∆gmt2/∆sgl1* to the lung environment, as this mutant exhibits a significant growth defect in physiological media (Fig. S6D). Thus, the rapid elimination of the *∆gmt2/∆sgl1* from the lung may be responsible for the insufficient stimulation of protective lung immunity.

Previous studies have suggested a correlation between small GXM filaments and immune response (49, 50); hence, we also considered whether this plays a role in the immune response that we observe. Our light scattering analysis showed that SG accumulation does not alter the GXM size pattern of either *∆gmt1* or *∆gmt2* (compare Fig. 9C to D and 9E to F). The three GXM peaks of the two single mutants do differ from each other; however, only *∆gmt1* shares the smallest peak with *∆sgl1* (at ~10 nm). Future work to define the material present in each peak may help explain the differences in behavior. Overall, we suspect that the size of GXM filaments is less important than the physical structure of this material in imparting protective immunity.

In considering the difference in the immune stimulation sufficient to induce protection only when the behavior of the vaccinating strains, we considered the possibility that additional immune mechanism(s) may be linked to the specific GXM structure of *∆sgl1*, in which the mannose M1 triad is missing. This is interesting because the same triad is missing in the *∆gmt1/∆sgl1* protective mutant, whereas it is present in the *∆gmt2/∆sgl1* mutant and WT strain. It may be that the absence of this M1 triad, together with the accumulation of SG, allows strains to stimulate the protective immune response in mice.

In our protective strains, *∆sgl1* and *∆gmt1/∆sgl1*, the usually most abundant M1 triad is missing, whereas M2 and to a lesser extent M3 are increased (Fig. 10B and C). Both of these triads are more highly xylosylated than M1; hence, we expected more xylose overall in GXM from these strains compared with WT or *∆gmt2/∆sgl1*. However, this expectation was not borne out by our compositional analysis, where the fraction of xylose is almost identical for the two protective strains and WT (Fig. S12). We cannot currently explain this discrepancy between the 1D-NMR and GC-MS data. It may be that the samples contain other carbohydrate contaminants. Further purification of the GXM samples and additional studies such as glycosyl linkage studies or 2-dimensional NMR analysis may elucidate this question. Recent findings indicate that the M2 triad is recognized by protective antibodies (25). However, it is unlikely that the M2 triad acts to regulate antibody stimulation: antibody production is not required for the protection conferred by *∆sgl1* because this protection still occurs in mice lacking B cells (22).

In contrast to the M2 triad, the absence of the M1 triad is crucial, suggesting that the presence of M1 may alter M2 immune stimulation. Perhaps the M2/M1 ratio may be important, and immune stimulation is sufficient to induce protection only when the M2 triad is more abundant than the M1 triad. The observation that IL-17A stimulation is decreased by *∆gmt2/∆sgl1*, where the M2/M1 ratio is lower than *∆gmt1/∆sgl1* or *∆sgl1*, may support this hypothesis. This stimulation is further decreased in WT, where the M1 triad is more abundant than the M2 triad (Fig. 10C). The different GXM structures, besides presenting different antigens, could also interfere with the exposure of other antigens present in the GXM, such as mannoproteins, or those antigens embedded in the cell wall, such as chitins and glucans, thus interfering with the immune stimulation.

Although Gmt1 and Gmt2 have the same biochemical activity, their function in *C. neoformans* is not interchangeable, as shown by the inability of Gmt2 overexpression to reverse the phenotypes of *∆gmt1* mutant (32, 35). In addition, although Gmt1 and Gmt2 mostly colocalize, they also appear in distinct sites of the secretory pathway (32). This suggests that they may play specialized roles in transporting GDP-mannose, which could potentially alter specific mannose polymerization in GXM and therefore change host responses to this material.

Another difference between GXM from different strains is in mannose acetylation, mediated in part by Cas1, which varies from 20 to 66% (51, 52). The partial loss of protection when mice were vaccinated with *∆cas1/∆sgl1* compared with *∆sgl1* alone suggests a possible relationship between this process and GXM-SG immune stimulation. Because of the relatively small change in protective phenotype, however, we did not examine γδ T cell stimulation by *∆cas1* or *∆cas1/∆sgl1*.

In conclusion, our results show that glucuronic acid and xylose modification of GXM is essential for the protective immunity conferred by cells that lack Sgl1 and therefore accumulate SGs. They also show that the distinct biological roles of Gmt1 and Gmt2 may extend to their influences on host response. Finally, we show that the M1 triad, which has one xylose and one glucuronic acid per three mannoses, is absent from GXM of strains that confer protection and may be therefore detrimental to the induction of protective immunity.

## MATERIALS AND METHODS

### Strains and culture conditions

The strains used in this study are wild-type (WT) *C. neoformans var. grubii* strain H99; *∆sgl1* mutant, various nucleotide-sugar transporter mutant strains (*∆uut1, ∆uxt1, ∆uxt2, ∆uxt1/∆uxt2, ∆gmt1,* and *∆gmt2*), and the *∆cas1* mutant, which was kindly provided by Francoise Dromer. The remaining mutants were generated in this study by CRISPR-Cas9 as described below. For all experiments, fungal strains were recovered from a −80°C freezer stock on YPD plates at 30°C for 3–4 days. An isolated colony was added to 10 mL of YPD broth and grown at 30°C for 16–18 h with shaking, washed with sterile PBS, counted on a hemocytometer, and resuspended in sterile PBS at the desired concentration and processed as described below. For the preparation of heat-killed strains, the desired concentration of yeast cells was resuspended in PBS and incubated in an 80°C heat block for 1 h. All heat-killed strains were confirmed to be dead by incubating the mixture on YPD plates at 30°C for 4 days and observing no growth.

### Strain construction and validation

Deletion of the *SGL1* gene was performed using a CRISPR with a short homology-directed repair system, as previously described (53). Briefly, overnight *C. neoformans* cultures were transferred to fresh YPD, with the initial inoculum of OD_600_ = 0.2, in a final volume of 100 mL. Cells were harvested after 5 h of growth, washed, and resuspended into electroporation buffer, where 700 ng of sg RNA targeting the *SGL1* gene was added together with 2 µg of HYGB-*SGL1* or 2 µg of NAT1-*SGL1* and 1 µg of Cas9. After electroporation, cells were transferred to liquid YPD and cultivated for 1 h, before selection on YPD plates supplemented with either 125 µg/mL of nourseothricin or 300 µg/mL of hygromycin. Initial validation of mutant strains was performed by PCR and eventually confirmed by Southern Blot analysis, using specific labeled probes with dCTP, [α−32P] (Revvity Health Sciences Inc). The complete list of restriction enzymes used for PCR can be found in Table S2.

### Growth curves

Fungal cells were grown in YPD overnight at 30°C under shaking. Then, the cells were washed with PBS, and 10^5^ cells/mL were added into 10 mL of Dulbecco’s Modified Eagle Medium (DMEM) at pH 4 or pH 7.4 at 37°C and 5% CO_2_. Samples of 100 µL were taken after 24, 48, or 72 h and plated in YPD plates for CFU quantification.

### Spot tests

Fungal cells were grown in YPD overnight at 30°C under shaking. Then, cells were washed twice in PBS, and 6 µL aliquots of serial 5-fold dilutions were plated (200, 500, 1,000, 2,000, or 10,000 cells) and grown at 37°C in solid media containing 0.01% SDS, 1.2 M NaCl, 1.2 M KCl, 0.05% Congo Red (CR), or 2% Calcofluor White (CFW). To test oxidative and nitrosative stress sensitivity, dilutions were spotted onto solid YPD medium (supplemented with 0.5 mM hydrogen peroxide [H_2_O_2_] or 0.5 mM sodium nitrite [NaNO_2_]).

### Urease activity

Cells were grown in Christensen’s Urea Agar for 5 days and then observed for the pink pigment formation. For the quantification of urease activity, cells were grown in Urea Broth according to Roberts et al. (54), and after 4 h of incubation, the absorbance at 560 nm was measured. Values are expressed as mean ± standard deviation from three independent experiments (*n* = 3) and analyzed by one-way ANOVA followed by Tukey’s post-test.

### Melanin production

To assess cell-associated melanin production, 5 µL of a 10^6^–10^4^ cells/mL solution was plated on agar plates containing 8 mg/mL KH_2_PO_4_, 2 mg/mL glucose, 2 mg/mL L-glycine, 1 µg/mL D-biotin, 1 µg/mL thiamine, 0.92 mg/mL MgSO_4_ 7H_2_O, and 0.4 mg/mL L-3,4-dihydrohyphenylalanine (L-DOPA; Sigma Aldrich).

### Phospholipase activity

*C. neoformans* strains were screened for extracellular phospholipase production by the method of Price et al. (55). Cultures were incubated at 37°C in egg yolk media, and the diameter of the zone of precipitate around the colonies was measured after 5 days. The ratio of the diameter of the colony to the total diameter of the colony plus the precipitation zone (Pz) was measured as an index of phospholipase activity. Values are expressed as mean ± standard deviation from six independent experiments (*n* = 6) and analyzed by one-way ANOVA followed by Tukey’s post-test.

### Capsule size determination

Cells were cultured in DMEM at 37°C with 5% CO₂ at pH 7.4 and harvested after 48 h of growth. Cell pellets were resuspended in PBS, and 10^5^ cells were used for India ink negative staining and observed under a Zeiss microscope. Pictures were taken using Zen Pro software. Total diameter (including capsule) (dt) and yeast cell diameter (dy) were measured for 50 cells of each strain. Capsule thickness (tc) was determined with the following equation: tc = 1/2(dt-dy) in micrometers. Values were expressed as mean ± standard and analyzed by one-way ANOVA followed by Tukey’s post-test.

### GXM extraction and purification

Extraction and purification of GXM were done by ultrafiltration as previously described (56). The amount of purified GXM from each strain was measured by phenol-sulfuric acid assay in 96-well plates as described previously by Masuko et al. (57).

### Lipid extraction and SGs analysis by liquid chromatography mass spectrometry (LC-MS)

Cells were cultivated in yeast nitrogen base (YNB) broth for 24 h at 30°C under shaking, and a pellet with 5 × 10^8^ cells was collected for lipid extraction. After the addition of a standard lipid mix from Avanti Polar Lipids (Alabaster, AL) for calibration purposes, extraction was performed using the Mandala method (58), followed by the Bligh and Dyer method (59). Samples were then analyzed by LC-MS. Ergosterol-3β-D-glucoside was detected as described previously (23). Data were analyzed on Thermo Xcalibur 2.2 Quan Browser software and normalized by inorganic phosphate content. For the determination of SG levels in culture media and GXM fraction, 10 mL aliquots were collected. Samples were dried, internal standards were added, and the lipids were extracted for LC-MS analysis. An aliquot of each sample was also used to measure total phosphates (Pi). Lipid results were normalized with nmol of Pi, as indicated.

### Mice

Male and female CBA/J 3- to 4-week-old mice purchased from The Jackson Laboratory were used in this study. All animals were housed at 3–4 animals per cage under specific-pathogen-free conditions and had access to food and water *ad libitum*. Mice were allowed 1 week to acclimate upon arrival before any procedures began.

### Survival studies and tissue burden culture analysis

All infections were performed intranasally. Fungal cells were grown overnight in YPD, counted, and resuspended in PBS. Animals were anesthetized with 60 µL xylazine/ketamine solution intraperitoneally (95 mg of ketamine and 5 mg of xylazine per kilogram of animal body weight). Inoculation of 5 × 10^5^ cells per animal in a volume of 20 µL was administered through nasal inhalation. Animals were checked daily, and those that had more than 20% wt loss or appeared moribund or in pain were euthanized using CO_2_ inhalation followed by cervical dislocation. For survival studies, 10 animals per group were monitored for 30 days after infection. For fungal burden analysis, the lungs and brains were collected from three animals per group after 3, 6, 9, 14, 21, and 30 days after infection. The homogenization of the lung and brain was performed with 10 mL of PBS using a Stomacher 80 blender (Seward, United Kingdom). The samples were then plated on YPD plates. Plates were incubated at a 30°C incubator, and after 48 h, CFUs were examined.

### Vaccination studies

For vaccination studies, 10 mice per group were used. Mice received PBS or 5 × 10^5^ *C. neoformans* live or dead mutant cells, as indicated, intranasally in a volume of 20 µL, 30 days before the challenge with the WT *C. neoformans* strain H99. After 30 days from the administration of the live mutant(s), mice were intranasally infected with 5 × 10^5^ WT H99 cells and followed for 60 days. Statistical analysis was performed using GraphPad Prism software version 9.0. Data are presented as means ± standard errors of the means. Statistical analysis was conducted using either a one-way ANOVA or a two-way repeated-measures ANOVA, followed by Tukey’s post hoc test for multiple comparisons. For survival curve assessments, the log-rank (Mantel-Cox) test was used, and the results are presented as mean percentages of survival. Fungal burden was analyzed as above, with samples also plated on YPD plates supplemented with either 125 µg/mL of nourseothricin or 300 µg/mL of hygromycin according to the mutant administered, to make sure that the fungus recovered from lungs or brains was the WT strain and not the mutants.

### *Ex vivo* splenocyte stimulation and IFNγ/IL-17A ELISA measurement

First, for plate preparation, 100 µL of the diluted anti-TCRγδ antibody (BioXCell; clone: UC7-13D5; concentration: 4 µg/mL) was incubated in a 96-well plate at 37°C and 5% CO2 for 4 h and washed twice with PBS to remove unbound antibody. Spleens were harvested from either infected or uninfected mice, washed, and processed for single-cell suspensions through a 70 µm pore filter. The cells were counted, and 10^5^ live γδ T cells were purified with a MACS separation kit (Miltenyi Biotec; catalog number 130-092-125) using an LD and MS column according to the manufacturer’s directions. Next, 10^5^ live *C. neoformans* cells of the desired strain or PBS as a control were added to the appropriate wells containing the γδ T cells in a 1:1 ratio. Seeded plates were incubated at 37°C and 5% CO_2_ for 5 days, and the supernatants were collected and stored at −80°C until ELISA was performed. The supernatants were divided and examined for IFNγ and IL-17A production using Legend Max Mouse IFNγ or IL-17A ELISA kits (BioLegend) following the manufacturer’s instructions.

### Macrophage phagocytosis assay

The phagocytosis assay was performed as described by Bryan et al. (60). Briefly, immortalized macrophages like cell line J774.1 (1 × 10^5^ per well) were plated in a 96-well plate, in 200 µL of DMEM supplemented with 10% of fetal bovine serum and 1% of penicillin/streptomycin. The cells were incubated overnight at 37°C, 5% CO_2_ to allow cell adhesion. The *C. neoformans* mutant strains were cultured in YNB medium. After overnight incubation at 30°C under agitation, the cells were washed twice with sterile PBS and counted. For macrophage activation and *C. neoformans* opsonization, a working solution was prepared using DMEM supplemented with LPS (0.3 ug/mL), IFN-γ (1 ng/mL), murine monoclonal 18B7 anti-GXM antibody (10 ug/mL), and the *C. neoformans* cells (1.25 × 10^5^ cell/well). The solution was incubated for 20 min, at 37°C under agitation, then washed twice with DMEM. Prior to infecting the macrophages, the cell monolayer was washed twice with DMEM without fetal bovine serum, and 200 µL of the working solution was added to each well. After 2 h of incubation at 37°C, 5% CO_2_, the cells were fixed and stained. At least 250 macrophage cells were counted per well. Ingested *C. neoformans* cells were distinguished from attached cells by the stained macrophage cell membrane surrounding the cryptococcal capsule. Phagocytic index is defined as the number of yeast cells ingested per number of macrophages per field.

### Analysis of *C. neoformans* surface architecture by 18B7 binding

*C. neoformans* was grown in Dulbecco’s Modified Eagle Medium (DMEM) high glucose, pyruvate (GIBCO, 11995–065) at 37°C, 5% CO_2_ for 48 h. The yeast cells were adjusted to 5 × 10^6^ cells, washed with PBS, and fixed in formalin 10% for 30 min. After washing with PBS, the fungal cells were blocked using 1% bovine serum albumin for 1 h at 37°C. Then, samples were incubated with 10 µg/mL of anti-GXM monoclonal antibody as in (61). Next, the samples were incubated with 10 µg/mL donkey anti-mouse IgG secondary, Alexa fluor 568 (Abcam, ab175472), and 5 µg/mL of calcofluor white (CFW, staining of chitin in the cell wall) (Sigma-Aldrich, 18909) for 30 min at 37°C, according to (62). The samples were mounted between the slide and coverslip and imaged at 100× magnification using a Zeiss Axio Observer microscope (Thornwood, NY). The images were processed using ImageJ, and the average fluorescence of at least 50 individual cells was measured. Mean fluorescence intensity values were normalized by subtracting the MFI of the acapsular strain *Δcap59*.

### Dynamic light scattering

The GXM samples obtained from the different strains were diluted 20-fold to 100-fold in filtered Milli-Q water to obtain a proper count rate for dynamic light scattering (DLS). DLS measurements were conducted on a Brookhaven Instruments Nanobrook Omni Particle Sizer equipped with a 40 mW red diode laser (nominal wavelength of 640 nm) at an angle of 90°. Experiments were conducted at 4°C, and each sample was measured at least three times using a 4 mL polystyrene cuvette to obtain the average and standard deviation. Particle size distributions were determined using non-negative least squares (NNLS) analysis. Peaks at very large sizes (>10 µm) were considered to be dust or large aggregates and were excluded from the analysis.

### Analysis by trimethylsilyl (TMS) derivatization and GC-MS of GXM

Glycosyl composition analysis was performed by gas chromatography-mass spectrometry (GC-MS) of the *O*-TMS methyl glycoside derivatives of the monosaccharides as modified from a method described previously by Coleman et al. (63). A 0.15–0.21-mg portion of each GXM sample was weighed out and transferred into a glass screw-top tube, and 20 µg of inositol was added as internal standard. In brief, the samples were lyophilized and then heated with methanolic 1 M HCl for 16 h at 80°C. After cooling and removing the solvent under a stream of nitrogen, the samples were re-N-acetylated with methanol, pyridine, and acetic anhydride and dried again. The sample was then derivatized with Tri-Sil (Thermo Scientific) at 80°C for 30 min. The TMS methyl glycosides were dried briefly under a stream of nitrogen and dissolved in hexane. The hexane was transferred to a new tube and dried; 150 µL of hexane was readded to the samples, and 1 µL was injected into the GS-MS. GC-MS analysis of the TMS residues was performed on an Agilent 7890A GC interfaced to a 5975C MSD, using a Supelco Equity-1 fused silica capillary column (30 m × 0.25 mm ID) employing a temperature gradient under a 50 to 1 split ratio mode.

### 1D-^1^H-NMR analysis

To obtain GXM for 1D-NMR, samples were pretreated as in (47, 64). Specifically, about 2–3 mg of each dried sample was dissolved in 2 mL of nano pure water, placed on ice, and ultrasonicated for a total of 10 min with the instrument alternatively on for 15 s and off for 15 s. Each treated sample was titrated on ice with concentrated ammonia to pH 11, followed by incubating at room temperature for 24 h. The samples were then dialyzed and lyophilized. Each treated sample was dissolved in 520 µL of D2O (99.96%) with 0.01 mg/mL of sodium trimethylsilyl propane sulfonate (DSS) internal standard (Cambridge Isotope Laboratories, Inc.). Each prepared sample was transferred into a 5 mm NMR tube. NMR data were obtained at 50°C on a Bruker Avance Neo 600 spectrometer with a TCI cryoprobe. ^1^H-NMR parameters: 60 s relaxation delay, 8992.8 Hz spectral width, 15323 data points, and 64 transients with a total recycle delay of 2.7 s between each transient. Prior to the Fourier transformation, the data were apodized with an exponential decay function with line broadening of 0.5 Hz, 90⁰ sine square, and zero-filled to 64 k points. The baselines were corrected automatically by subtracting a third-order polynomial. The spectra were processed and analyzed with Mestre Nova (version ×64).

### Statistical analysis and study design

All statistical analyses were performed using GraphPad Prism 9 software. The sample size, statistical analysis, and statistical significance are all described for each figure in the figure captions. No data were excluded from the statistical analysis. One to three experimental replicates were carried out for all data sets, and when applicable, a representative figure is shown for each experiment. For *in vivo* survival studies, 10 mice per group were used for statistical power and to reduce the number of mice used throughout the study. As mentioned above, statistical analysis was conducted using either a one-way ANOVA or a two-way repeated-measures ANOVA, followed by Tukey’s post hoc test for multiple comparisons. For survival curve assessments, the log-rank (Mantel-Cox) test was used, and the results are presented as mean percentages of survival.

## SUPPLEMENTAL MATERIAL

Supplemental material mbio.00984-25-s0001.pdf Supplemental tables and figures.

ASM does not own the copyrights to Supplemental Material that may be linked to, or accessed through, an article. The authors have granted ASM a non-exclusive, world-wide license to publish the Supplemental Material files. Please contact the corresponding author directly for reuse.

## References

[1] Rajasingham R, Smith RM, Park BJ, Jarvis JN, Govender NP, Chiller TM, Denning DW, Loyse A, Boulware DR. 2017. Global burden of disease of HIV-associated cryptococcal meningitis: an updated analysis. Lancet Infect Dis 17:873–881. doi:10.1016/S1473-3099(17)30243-8 28483415 PMC5818156

[2] Stott KE, Loyse A, Jarvis JN, Alufandika M, Harrison TS, Mwandumba HC, Day JN, Lalloo DG, Bicanic T, Perfect JR, Hope W. 2021. Cryptococcal meningoencephalitis: time for action. Lancet Infect Dis 21:e259–e271. doi:10.1016/S1473-3099(20)30771-4 33872594

[3] Walukaga S, Fieberg A, Musubire A, Tugume L, Ssebambulidde K, Kagimu E, Kasibante J, Rutakingirwa MK, Mpoza E, Gakuru J, Akampurira A, Jjunju S, Mwesigye J, Muzoora C, Nuwagira E, Bangdiwala AS, Williams DA, Rhein J, Meya DB, Boulware DR, Hullsiek KH, Rajasingham R, ASTRO team. 2024. The evolution of HIV-associated cryptococcal meningitis in Uganda from 2010 to 2022. Med Mycol 63:myae115. doi:10.1093/mmy/myae115 39779301 PMC11718514

[4] Engelthaler DM, Casadevall A. 2019. On the emergence of Cryptococcus gattii in the Pacific northwest: ballast tanks, tsunamis, and black swans. MBio 10:e02193-19. doi:10.1128/mBio.02193-19 31575770 PMC6775458

[5] Denning DW. 2024. Global incidence and mortality of severe fungal disease. Lancet Infect Dis 24:e428–e438. doi:10.1016/S1473-3099(23)00692-8 38224705

[6] Meena P, Bhargava V, Singh K, Sethi J, Prabhakar A, Panda S. 2023. Cryptococcosis in kidney transplant recipients: current understanding and practices. World J Nephrol 12:120–131. doi:10.5527/wjn.v12.i5.120 38230297 PMC10789088

[7] Wald-Dickler N, She R, Blodget E. 2017. Cryptococcal disease in the solid organ transplant setting: review of clinical aspects with a discussion of asymptomatic cryptococcal antigenemia. Curr Opin Organ Transplant 22:307–313. doi:10.1097/MOT.0000000000000426 28562416

[8] Lin X, Heitman J. 2006. The biology of the Cryptococcus neoformans species complex. Annu Rev Microbiol 60:69–105. doi:10.1146/annurev.micro.60.080805.142102 16704346

[9] Enoch DA, Ludlam HA, Brown NM. 2006. Invasive fungal infections: a review of epidemiology and management options. J Med Microbiol 55:809–818. doi:10.1099/jmm.0.46548-0 16772406

[10] Del Poeta M, Wormley FL Jr, Lin X. 2023. Host populations, challenges, and commercialization of cryptococcal vaccines. PLoS Pathog 19:e1011115. doi:10.1371/journal.ppat.1011115 36757929 PMC9910758

[11] Zhai B, Wozniak KL, Masso-Silva J, Upadhyay S, Hole C, Rivera A, Wormley FL, Lin X. 2015. Development of protective inflammation and cell-mediated immunity against Cryptococcus neoformans after exposure to hyphal mutants. MBio 6:e01433-15. doi:10.1128/mBio.01433-15 26443458 PMC4611043

[12] Wang R, Oliveira LVN, Hester MM, Carlson D, Christensen D, Specht CA, Levitz SM. 2024. Protection against experimental cryptococcosis elicited by Cationic Adjuvant Formulation 01-adjuvanted subunit vaccines. PLoS Pathog 20:e1012220. doi:10.1371/journal.ppat.1012220 38976694 PMC11257399

[13] Hole CR, Wager CML, Castro-Lopez N, Campuzano A, Cai H, Wozniak KL, Wang Y, Wormley FL Jr. 2019. Induction of memory-like dendritic cell responses in vivo. Nat Commun 10:2955. doi:10.1038/s41467-019-10486-5 31273203 PMC6609631

[14] Van Dyke MCC, Chaturvedi AK, Hardison SE, Leopold Wager CM, Castro-Lopez N, Hole CR, Wozniak KL, Wormley FL Jr. 2017. Induction of broad-spectrum protective immunity against disparate Cryptococcus serotypes. Front Immunol 8:1359. doi:10.3389/fimmu.2017.01359 29163469 PMC5670106

[15] Li Y, Pham T, Hipsher K, Lee CWJ, Jiao J, Penninger JM, Kronstad JW, Fan Y, Zhao Y, Ambati S, Meagher RB, Xie X, Lin X. 2025. Identification of a protective antigen reveals the trade-off between iron acquisition and antigen exposure in a global fungal pathogen. Proc Natl Acad Sci USA 122:e2420898122. doi:10.1073/pnas.2420898122 39946532 PMC11848283

[16] Specht CA, Lam WC, Hester MM, Lourenco D, Levitz SM, Lodge JK, Upadhya R. 2024. Chitosan-deficient Cryptococcus as whole-cell vaccines. Methods Mol Biol 2775:393–410. doi:10.1007/978-1-0716-3722-7_27 38758333 PMC11521572

[17] Specht CA, Wang R, Oliveira LVN, Hester MM, Gomez C, Mou Z, Carlson D, Lee CK, Hole CR, Lam WC, Upadhya R, Lodge JK, Levitz SM. 2024. Immunological correlates of protection mediated by a whole organism, Cryptococcus neoformans, vaccine deficient in chitosan. MBio 15:e0174624. doi:10.1128/mbio.01746-24 38980038 PMC11323574

[18] Ueno K, Tsuge S, Shimizu K, Miyazaki Y. 2023. Promising whole-cell vaccines against cryptococcosis. Microbiol Immunol 67:211–223. doi:10.1111/1348-0421.13056 36786396

[19] Rella A, Mor V, Farnoud AM, Singh A, Shamseddine AA, Ivanova E, Carpino N, Montagna MT, Luberto C, Del Poeta M. 2015. Role of Sterylglucosidase 1 (Sgl1) on the pathogenicity of Cryptococcus neoformans: potential applications for vaccine development. Front Microbiol 6:836. doi:10.3389/fmicb.2015.00836 26322039 PMC4531891

[20] Normile TG, Bryan AM, Del Poeta M. 2020. Animal models of Cryptococcus neoformans in identifying immune parameters associated with primary infection and reactivation of latent infection. Front Immunol 11:581750. doi:10.3389/fimmu.2020.581750 33042164 PMC7522366

[21] Normile TG, Chu TH, Sheridan BS, Del Poeta M. 2022. Vaccine protection by Cryptococcus neoformans Δsgl1 is mediated by γδ T cells via TLR2 signaling. Mucosal Immunol 15:1416–1430. doi:10.1038/s41385-022-00570-3 36229573 PMC9705245

[22] Normile TG, Rella A, Del Poeta M. 2021. Cryptococcus neoformans Δsgl1 vaccination requires either CD4^+^ or CD8^+^ T cells for complete host protection. Front Cell Infect Microbiol 11:739027. doi:10.3389/fcimb.2021.739027 34568097 PMC8455912

[23] Colombo AC, Rella A, Normile T, Joffe LS, Tavares PM, de S Araújo GR, Frases S, Orner EP, Farnoud AM, Fries BC, Sheridan B, Nimrichter L, Rodrigues ML, Del Poeta M. 2019. Cryptococcus neoformans glucuronoxylomannan and sterylglucoside are required for host protection in an animal vaccination model. MBio 10:e02909-18. doi:10.1128/mBio.02909-18 30940711 PMC6445945

[24] Kuttel MM, Casadevall A, Oscarson S. 2020. Cryptococcus neoformans capsular GXM conformation and epitope presentation: a molecular modelling study. Molecules 25:2651. doi:10.3390/molecules25112651 32517333 PMC7321252

[25] Hargett AA, Azurmendi HF, Crawford CJ, Wear MP, Oscarson S, Casadevall A, Freedberg DI. 2024. The structure of a C. neoformans polysaccharide motif recognized by protective antibodies: a combined NMR and MD study. Proc Natl Acad Sci U S A 121:e2315733121. doi:10.1073/pnas.2315733121 38330012 PMC10873606

[26] Fu Y, Huang X, Zhou Z. 2020. Insight into the assembling mechanism of Cryptococcus capsular glucuronoxylomannan based on molecular dynamics simulations. ACS Omega 5:29351–29356. doi:10.1021/acsomega.0c04164 33225166 PMC7676341

[27] Wang ZA, Li LX, Doering TL. 2018. Unraveling synthesis of the cryptococcal cell wall and capsule. Glycobiology 28:719–730. doi:10.1093/glycob/cwy030 29648596 PMC6142866

[28] Decote-Ricardo D, LaRocque-de-Freitas IF, Rocha JDB, Nascimento DO, Nunes MP, Morrot A, Freire-de-Lima L, Previato JO, Mendonça-Previato L, Freire-de-Lima CG. 2019. Immunomodulatory role of capsular polysaccharides constituents of Cryptococcus neoformans. Front Med 6:129. doi:10.3389/fmed.2019.00129 PMC6593061 31275938

[29] Devi SJ, Schneerson R, Egan W, Ulrich TJ, Bryla D, Robbins JB, Bennett JE. 1991. Cryptococcus neoformans serotype A glucuronoxylomannan-protein conjugate vaccines: synthesis, characterization, and immunogenicity. Infect Immun 59:3700–3707. doi:10.1128/iai.59.10.3700-3707.1991 1716613 PMC258941

[30] Datta K, Pirofski L. 2006. Towards a vaccine for Cryptococcus neoformans: principles and caveats. FEMS Yeast Res 6:525–536. doi:10.1111/j.1567-1364.2006.00073.x 16696648

[31] Casadevall A, Pirofski L. 2005. Insights into mechanisms of antibody-mediated immunity from studies with Cryptococcus neoformans. Curr Mol Med 5:421–433. doi:10.2174/1566524054022567 15977998

[32] Wang ZA, Griffith CL, Skowyra ML, Salinas N, Williams M, Maier EJ, Gish SR, Liu H, Brent MR, Doering TL. 2014. Cryptococcus neoformans dual GDP-mannose transporters and their role in biology and virulence. Eukaryot Cell 13:832–842. doi:10.1128/EC.00054-14 24747214 PMC4054277

[33] Doering TL. 2009. How sweet it is! Cell wall biogenesis and polysaccharide capsule formation in Cryptococcus neoformans. Annu Rev Microbiol 63:223–247. doi:10.1146/annurev.micro.62.081307.162753 19575556 PMC2880894

[34] Li LX, Rautengarten C, Heazlewood JL, Doering TL. 2018. UDP-glucuronic acid transport is required for virulence of Cryptococcus neoformans. MBio 9:e02319-17. doi:10.1128/mBio.02319-17 29382737 PMC5790919

[35] Cottrell TR, Griffith CL, Liu H, Nenninger AA, Doering TL. 2007. The pathogenic fungus Cryptococcus neoformans expresses two functional GDP-mannose transporters with distinct expression patterns and roles in capsule synthesis. Eukaryot Cell 6:776–785. doi:10.1128/EC.00015-07 17351078 PMC1899245

[36] Li LX, Rautengarten C, Heazlewood JL, Doering TL. 2018. Xylose donor transport is critical for fungal virulence. PLoS Pathog 14:e1006765. doi:10.1371/journal.ppat.1006765 29346417 PMC5773217

[37] Cox GM, McDade HC, Chen SCA, Tucker SC, Gottfredsson M, Wright LC, Sorrell TC, Leidich SD, Casadevall A, Ghannoum MA, Perfect JR. 2001. Extracellular phospholipase activity is a virulence factor for Cryptococcus neoformans . Mol Microbiol 39:166–175. doi:10.1046/j.1365-2958.2001.02236.x 11123698

[38] Hamed MF, Araújo GR de S, Munzen ME, Reguera-Gomez M, Epstein C, Lee HH, Frases S, Martinez LR. 2023. Phospholipase B is critical for Cryptococcus neoformans survival in the central nervous system. MBio 14:e0264022. doi:10.1128/mbio.02640-22 36786559 PMC10127605

[39] Toplis B, Bosch C, Schwartz IS, Kenyon C, Boekhout T, Perfect JR, Botha A. 2020. The virulence factor urease and its unexplored role in the metabolism of Cryptococcus neoformans. FEMS Yeast Res 20:foaa031. doi:10.1093/femsyr/foaa031 32490521 PMC7592176

[40] Baker RP, Liu AZ, Casadevall A. 2024. Cell wall melanin impedes growth of the Cryptococcus neoformans polysaccharide capsule by sequestering calcium. Proc Natl Acad Sci U S A 121:e2412534121. doi:10.1073/pnas.2412534121 39259590 PMC11420191

[41] Mednick AJ, Nosanchuk JD, Casadevall A. 2005. Melanization of Cryptococcus neoformans affects lung inflammatory responses during cryptococcal infection. Infect Immun 73:2012–2019. doi:10.1128/IAI.73.4.2012-2019.2005 15784542 PMC1087470

[42] Nosanchuk JD, Rosas AL, Casadevall A. 1998. The antibody response to fungal melanin in mice. J Immunol 160:6026–6031. doi:10.4049/jimmunol.160.12.6026 9637518

[43] Liu S, Youngchim S, Zamith-Miranda D, Nosanchuk JD. 2021. Fungal melanin and the mammalian immune system. J Fungi (Basel) 7:264. doi:10.3390/jof7040264 33807336 PMC8066723

[44] Normile TG, Del Poeta M. 2022. Three models of vaccination strategies against cryptococcosis in immunocompromised hosts using heat-killed Cryptococcus neoformans Δsgl1. Front Immunol 13:868523. doi:10.3389/fimmu.2022.868523 35615354 PMC9124966

[45] De Jesus M, Chow S-K, Cordero RJB, Frases S, Casadevall A. 2010. Galactoxylomannans from Cryptococcus neoformans varieties neoformans and grubii are structurally and antigenically variable. Eukaryot Cell 9:1018–1028. doi:10.1128/EC.00268-09 20061411 PMC2901672

[46] Doering TL. 2000. How does Cryptococcus get its coat? Trends Microbiol 8:547–553. doi:10.1016/s0966-842x(00)01890-4 11115750

[47] Cherniak R, Valafar H, Morris LC, Valafar F. 1998. Cryptococcus neoformans chemotyping by quantitative analysis of 1H nuclear magnetic resonance spectra of glucuronoxylomannans with a computer-simulated artificial neural network. Clin Diagn Lab Immunol 5:146–159. doi:10.1128/CDLI.5.2.146-159.1998 9521136 PMC121351

[48] Fries BC, Goldman DL, Cherniak R, Ju R, Casadevall A. 1999. Phenotypic switching in Cryptococcus neoformans results in changes in cellular morphology and glucuronoxylomannan structure. Infect Immun 67:6076–6083. doi:10.1128/IAI.67.11.6076-6083.1999 10531269 PMC96995

[49] McFadden DC, De Jesus M, Casadevall A. 2006. The physical properties of the capsular polysaccharides from Cryptococcus neoformans suggest features for capsule construction. J Biol Chem 281:1868–1875. doi:10.1074/jbc.M509465200 16278213

[50] Fonseca FL, Nohara LL, Cordero RJB, Frases S, Casadevall A, Almeida IC, Nimrichter L, Rodrigues ML. 2010. Immunomodulatory effects of serotype B glucuronoxylomannan from Cryptococcus gattii correlate with polysaccharide diameter. Infect Immun 78:3861–3870. doi:10.1128/IAI.00111-10 20547742 PMC2937472

[51] Bacon BE, Cherniak R, Kwon-Chung KJ, Jacobson ES. 1996. Structure of the O-deacetylated glucuronoxylomannan from Cryptococcus neoformans Cap70 as determined by 2D NMR spectroscopy. Carbohydr Res 283:95–110. doi:10.1016/0008-6215(95)00397-5 8901265

[52] Janbon G, Himmelreich U, Moyrand F, Improvisi L, Dromer F. 2001. Cas1p is a membrane protein necessary for the O-acetylation of the Cryptococcus neoformans capsular polysaccharide. Mol Microbiol 42:453–467. doi:10.1046/j.1365-2958.2001.02651.x 11703667

[53] Huang MY, Joshi MB, Boucher MJ, Lee S, Loza LC, Gaylord EA, Doering TL, Madhani HD. 2022. Short homology-directed repair using optimized Cas9 in the pathogen Cryptococcus neoformans enables rapid gene deletion and tagging. Genetics 220:iyab180. doi:10.1093/genetics/iyab180 34791226 PMC8733451

[54] Roberts GD, Horstmeier CD, Land GA, Foxworth JH. 1978. Rapid urea broth test for yeasts. J Clin Microbiol 7:584–588. doi:10.1128/jcm.7.6.584-588.1978 353068 PMC275077

[55] Price MF, Wilkinson ID, Gentry LO. 1982. Plate method for detection of phospholipase activity in Candida albicans. Sabouraudia 20:7–14. doi:10.1080/00362178285380031 7038928

[56] Nimrichter L, Frases S, Cinelli LP, Viana NB, Nakouzi A, Travassos LR, Casadevall A, Rodrigues ML. 2007. Self-aggregation of Cryptococcus neoformans capsular glucuronoxylomannan is dependent on divalent cations. Eukaryot Cell 6:1400–1410. doi:10.1128/EC.00122-07 17573547 PMC1951138

[57] Masuko T, Minami A, Iwasaki N, Majima T, Nishimura S-I, Lee YC. 2005. Carbohydrate analysis by a phenol-sulfuric acid method in microplate format. Anal Biochem 339:69–72. doi:10.1016/j.ab.2004.12.001 15766712

[58] Mandala SM, Thornton RA, Frommer BR, Curotto JE, Rozdilsky W, Kurtz MB, Giacobbe RA, Bills GF, Cabello MA, Martín I. 1995. The discovery of australifungin, a novel inhibitor of sphinganine N-acyltransferase from Sporormiella australis. Producing organism, fermentation, isolation, and biological activity. J Antibiot (Tokyo) 48:349–356. doi:10.7164/antibiotics.48.349 7797434

[59] Bligh EG, Dyer WJ. 1959. A rapid method for total lipid extraction and purification. Can J Biochem Physiol 37:911–917. 13671378 10.1139/o59-099

[60] Bryan AM, Farnoud AM, Mor V, Del Poeta M. 2014. Macrophage cholesterol depletion and its effect on the phagocytosis of Cryptococcus neoformans. J Vis Exp 94:e52432. doi:10.3791/52432-v PMC4396961 25549203

[61] Casadevall A, Cleare W, Feldmesser M, Glatman-Freedman A, Goldman DL, Kozel TR, Lendvai N, Mukherjee J, Pirofski LA, Rivera J, Rosas AL, Scharff MD, Valadon P, Westin K, Zhong Z. 1998. Characterization of a murine monoclonal antibody to Cryptococcus neoformans polysaccharide that is a candidate for human therapeutic studies. Antimicrob Agents Chemother 42:1437–1446. doi:10.1128/AAC.42.6.1437 9624491 PMC105619

[62] Rodrigues ML, Alvarez M, Fonseca FL, Casadevall A. 2008. Binding of the wheat germ lectin to Cryptococcus neoformans suggests an association of chitinlike structures with yeast budding and capsular glucuronoxylomannan. Eukaryot Cell 7:602–609. doi:10.1128/EC.00307-07 18039942 PMC2292635

[63] Coleman CM, Auker KM, Killday KB, Azadi P, Black I, Ferreira D. 2019. Arabinoxyloglucan oligosaccharides may contribute to the antiadhesive properties of porcine urine after cranberry consumption. J Nat Prod 82:589–605. doi:10.1021/acs.jnatprod.8b01043 30873836

[64] Kumar P, Heiss C, Santiago-Tirado FH, Black I, Azadi P, Doering TL. 2014. Pbx proteins in Cryptococcus neoformans cell wall remodeling and capsule assembly. Eukaryot Cell 13:560–571. doi:10.1128/EC.00290-13 24585882 PMC4060484

