# Supplementary material for: Role of glucuronoxylomannan and steryl glucosides in protecting against cryptococcosis
Source: mBio. 2025 Apr 29;16(6):e00984-25. doi: 10.1128/mbio.00984-25 (PMC12153286; doi:10.1128/mbio.00984-25)
Supplement: Supplemental material — Supplemental tables and figures. [file mbio.00984-25-s0001.pdf]

Supplementary Material

Role of glucuronoxylomannan and sterylglucosides in protecting against cryptococcosis.

Gabriel Soares Matos[1]*, Samyr M. Querobino[1]*, Veronica S. Brauer[1], Luna S. Joffe[1], Nivea Pereira de Sa[1], Caroline Mota Fernandes[1], Deveney DaSilva[1], Vanessa A. da Silva[1], Marinaldo Pacífico Cavalcanti Neto[1], Tyler Normile[1], Hengwei Zhu[2], Surita R. Bhatia[2], Li Tan[3], Parastoo Azadi[3], Christian Heiss[3], Tamara L. Doering[4], and Maurizio Del Poeta[1,5,6,#].

[1]Department of Microbiology and Immunology, Stony Brook University, Stony Brook, New York, USA; [2]Department of Chemistry, Stony Brook University, Stony Brook, New York, USA; [3]Complex Carbohydrate Research Center, University of Georgia, Athens, GA, USA; [4]Department of Molecular Microbiology, Washington University School of Medicine, St. Louis, MO, USA; [5]Division of Infectious Diseases, School of Medicine, Stony Brook University, Stony Brook, New York, USA; [6]Veterans Affairs Medical Center, Northport, New York, USA.

*** These authors contributed equally**

Keywords: sterylglucosides, glucuronoxylomannan, xylose, mannose, glucuronic acid, *Cryptococcus neoformans*, fungal infection, vaccine, immunity

Running Title: Role GXM and SGs on cryptococcal vaccination

[#] Corresponding author:
Maurizio Del Poeta, M.D.
Department of Microbiology and Immunology
Stony Brook University, 150 Life Science Building, Stony Brook, NY 11794.
E-mail: maurizio.delpoeta@stonybrook.edu; Tel: +1 (631) 632-4024

Supplementary Table 1. *Cryptococcus neoformans* strains used in this study

C. neoformans **strains**	**Genotype**	**Origin**
H99	Wild-Type	ATCC-208821
Δ*sgl1*	*Δsgl1::NAT1*	1
Δ*uut1*	*Δuut1::NAT1*	2
Δ*uut1*/Δsgl1	*Δuut1::NAT1 Δsgl1::hyg^R*	This Study
Δ*uxt1*	*Δuxt1::G418^R*	3
Δ*uxt1*/Δ*sgl1*	*Δuxt1::G418^R Δsgl1::hyg^R*	This Study
Δ*uxt2*	*Δuxt2::NAT1*	3
Δ*uxt2*/Δ*sgl1*	*Δuxt2::NAT1 Δsgl1::hyg^R*	This Study
Δ*uxt1*/Δ*uxt2*	*Δuxt1::G418^R Δuxt2::NAT1*	3
Δ*uxt1*/Δ*uxt2/Δsgl1*	*Δuxt1::G418^R Δuxt2::NAT1 Δsgl1:: hyg^R*	This Study
Δ*gmt1*	*Δgmt1::hyg^R*	4
Δ*gmt1*/Δ*sgl1*	*Δgmt1::hyg^R Δsgl1::NAT1*	This Study
Δ*gmt2*	*Δgmt2::hyg^R*	4
Δ*gmt2*/Δ*sgl1*	*Δgmt2::hyg^R Δsgl1::NAT1*	This Study
Δ*cas1*	*Δcas1::hyg^R*	5
Δ*cas1/Δsgl1*	*Δcas1::hyg^R Δsgl1::NAT1*	This Study

1. Rella A., *et al.* Role of Sterylglucosidase 1 (Sgl1) on the pathogenicity of *Cryptococcus neoformans*: potential applications for vaccine development. Front Microbiol. 2015 Aug 11;6:836.
2. Li LX, Rautengarten C, Heazlewood JL, Doering TL. UDP-Glucuronic Acid Transport Is Required for Virulence of *Cryptococcus neoformans*. mBio. 2018 Jan 30;9(1):e02319-17
3. Li LX, Rautengarten C, Heazlewood JL, Doering TL (2018) Xylose donor transport is critical for fungal virulence. PLOS Pathogens 14(1): e1006765.
4. Wang ZA, *et al*. *Cryptococcus neoformans* dual GDP-mannose transporters and their role in biology and virulence. Eukaryot Cell. 2014 Jun;13(6):832-42.
5. Janbon G, *et al*. 2001. Cas1p is a membrane protein necessary for the O-acetylation of the *Cryptococcus neoformans* capsular polysaccharide. Mol Microbiol 42:453–467.

Supplementary Table 2. List of primers used in this study

List of primers				
SGL1 Deletion (Plasmid)	Pair 1	knockout cassette	5’UTR-F	GTCAAGCTAAGAGCTCCATTTGATCAGCGGGATTCT
			5’UTR-R	TCCACTCCGAACTAGTATCGCGTAAACGAAGAGGTG
			3’UTR-F	GTCAAGCTAATCTAGAAGCCCATTCTGGTTGTTCTG
			3’UTR-R	ACATCACACTTCTAGATTTAGCGAGCCACGTTTTCT
	Pair 2	5'UTR Probe	Fw	CCGCTTCATCGTCCGACATA
			Rv	CAGCGGCTGACCTTGGAATA
	Pair 3	Gene probe	Fw	GGTGAGTGGATGTTCGAGAGG
			Rv	CCCCTTGTCCAACCCCATTT
Crispr-Cas9 system	Pair4	M13	Fw	TGTAAAACGACGGCCAGTG
			Rv	GCGGATAACAATTTCACACAGG
	Pair 5	C8573	Fw	AATTGGAGCTCCACCGCG
		c8574	Rv	GGGAACAAAAGCTGGGTACC
GMT1 Deletion	Pair 6	Deletion cassete	Fw	TTTCTCTTGCATCTCAACTTTTCCCCCAAAGTAATAACCATCTCGGCACCGCA TGGTGTGCGCTGAGTGT
			Rv	CGTGAATCATCACCAAACGTAGTCCTCGTCTCTTCCATTCCAAAAGCATCTC ACAGTTAAATTGCTAACG
	Pair 7	SgRNA	Fw	TCTCGACTACGGCAAGGACGGTTTTAGAGCTAGAAATAGCAAGTT
			Rv	CGTCCTTGCCGTAGTCGAGACAACAGTATACCCTGCCGGTG
	Pair 8	3'UTR probe	Fw	ACCGTGAAGAAGCTCGGTTT
			Rv	ACAAGTTCCGTACCGCCAAT
	Pair 9	Gene probe	Fw	GGACTTTTTGGTGCACGGAC
			Rv	CAATGACCGCTGTTATGCGG
GMT2 deletion	Pair 10	Deletion cassete	Fw	GAAAGTTACACCTTTCCACATCTTTTCGTTTCTCTATCCACGCCAAAGCCGCA TGGTGTGCGCTGAGTGT
			Rv	GGAAAGAAGAAAAAAAAAAAGACACCACACAGCACAGACATTATTACGCC TCACAGTTAAATTGCTAACG
	Pair 11	SgRNA	Fw	CCTCTGGGTCTCTCTCCCAGGTTTTAGAGCTAGAAATAGCAAGTT
			Rv	CTGGGAGAGAGACCCAGAGGCAACAGTATACCCTGCCGGTG
	Pair 12	3'UTR probe	Fw	TTGTTCGTGGATGGTGCTGA
			Rv	GATGACGGATGAACCGACCA
	Pair 13	Gene probe	Fw	CCAAAGCGTGGTTTCCAGTG
			Rv	TATGAGATGAAGACGGCGGC
SGL1 Deletion (Crispr)	Pair 14	Deletion cassete	Fw	CGTTAGCAATTTAACTGTGAGTGTGTCAAAGGAAGTCAATATAATGGGCAA GCGCTGATTAACTTATCTC
			Rv	GAGATAAGTTAATCAGCGCTTGCCCATTATATTGACTTCCTTTGACACACTC ACAGTTAAATTGCTAACG
	Pair 15	SgRNA	Fw	ACCAGAAGTCTCTCCTGTCAGTTTTAGAGCTAGAAATAGCAAGTT
			Rv	TGACAGGAGAGACTTCTGGTCAACAGTATACCCTGCCGGTG
	Pair 16	3'UTR probe	Fw	TGGAAGCCCATTCTGGTTGT
			Rv	CTTCTTGCTTTCTGACCCGC
	Pair 16	Gene probe	Fw	TGGACCGATGGCTGTGTAGA
			Rv	TGCTGTAGGCATAGGCTTGG
CAS1 Deletion	Pair 18	Deletion cassete	Fw	CATCCTGCAGCCATTGGGCAGTTTATAGCCAAGGAGGGCAGAGGTCCGAA GCATGGTGTGCGCTGAGTGT
			Rv	TAATGAAGGTATATCAAATTCTCCTCTAAAGTAAAAAAATAAATCAATCTTC ACAGTTAAATTGCTAACG
	Pair 19	SgRNA	Fw	CCGCAAAGCTCAACCCTCTCGTTTTAGAGCTAGAAATAGCAAGTT
			Rv	GAGAGGGTTGAGCTTTGCGGCAACAGTATACCCTGCCGGTG
	Pair 20	3'UTR probe	Fw	CTGATGGCGTCGTAGTCTGG
			Rv	CAGCGCTTCACCGATTCTTG
	Pair 21	Gene probe	Fw	CAGCGAGTTGTCATGGTCCT
			Rv	AGACGCGGTAACTCAACCAG

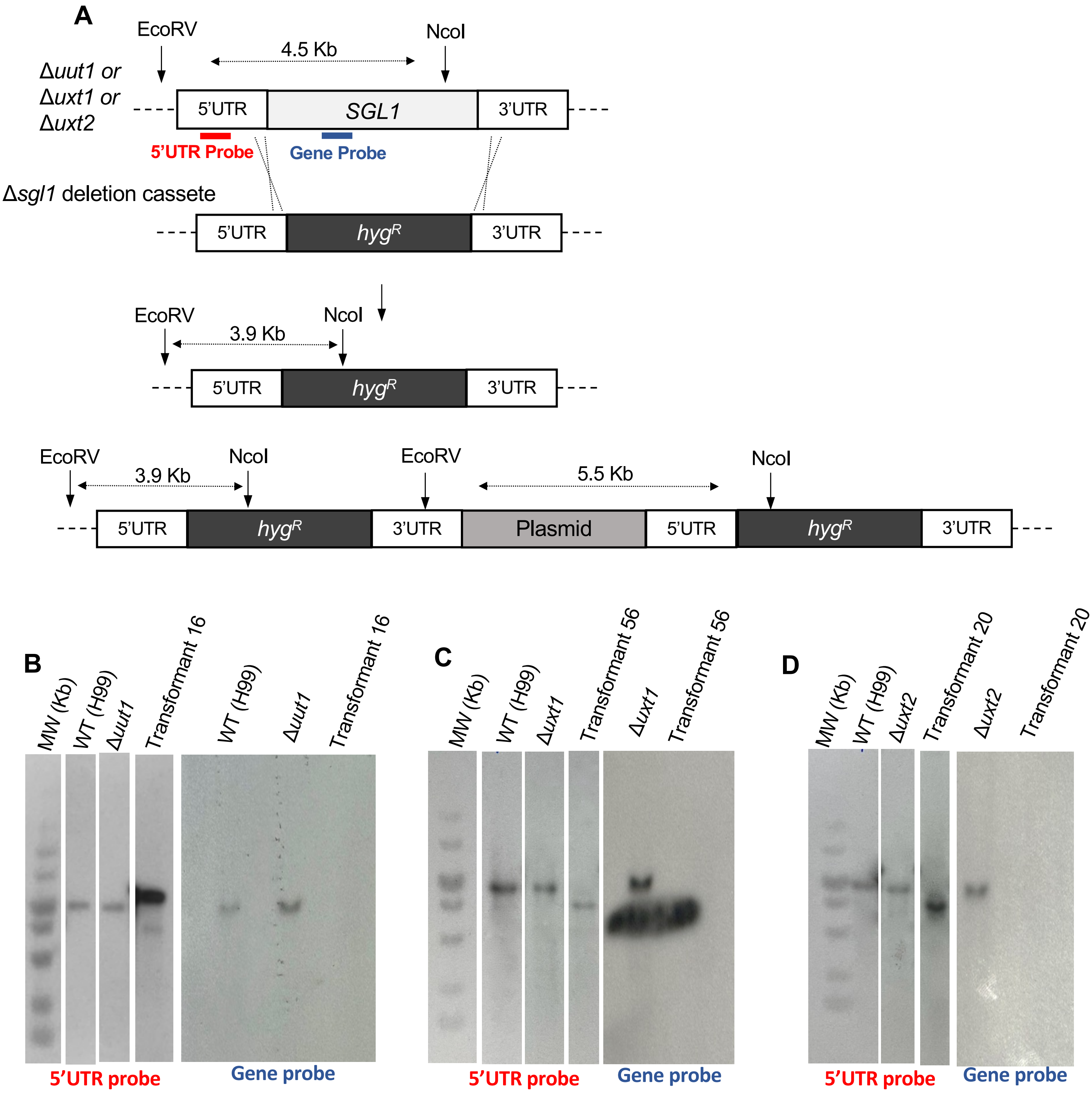

Supplementary Figure 1. Deletion of *SGL1* gene in *Δuut1, Δuxt1* or *Δuxt2*. (A) Strategy for the deletion of *SGL1* gene using a Hygromycin (HygR) cassette. Restriction enzymes and the size of the DNA fragment are represented. B, C and D) Southern blot analysis of genomic DNA digested with EcoRV and NcoI using the 5′-UTR probe or the gene probe, which are represented by a red and blue bar respectively in panel A. (B) Transformant #16 showed an expected band of 5.5 and 3.9 Kb when the 5'-UTR probe was used and no band when gene probe was used. Transformant #16 was named *Δuut1/Δsgl1* mutant. C) Transformant #56 showed an expected band of 3.9 Kb when the 5'-UTR was used and no apparent band when the gene probe was used. Transformant #56 was named *Δuxt1/Δsgl1* mutant. D) Transformant #20 showed an expected band of 3.9 Kb when 5′-UTR probe was used and no band when the gene probe was used. Transformant #20 was named *Δuxt2/Δsgl1* mutant.

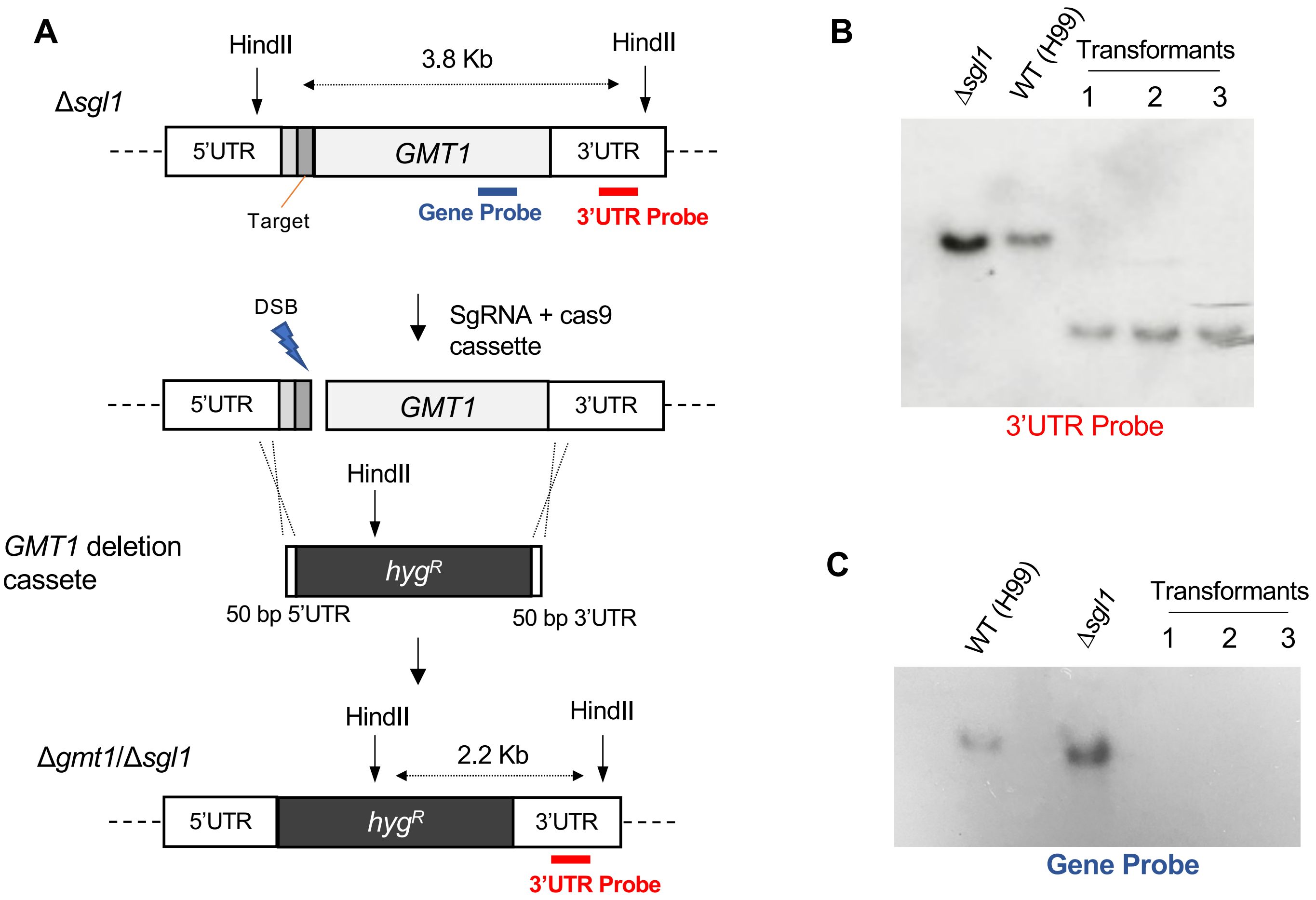

Supplementary Figure 2. Deletion of the *GMT1* gene in the *Δsgl1* background. (A) Diagram of the targeted deletion of *GMT1* gene via CRISPR-Cas9-mediated homologous recombination in the background of Δ*sgl1* strain. The Cas9 target site is exemplified in dark gray and the sites of homologous recombination with the selective marker are represented in white. (B) The transformants were analyzed by Southern Blot analysis, by digesting the genomic DNA with Hind II restriction enzyme and by subjecting the blot to 3'-UTR probe. The control WT (H99) and Δ*sgl1* showed a band of 3.8 Kb, whereas transformant #1, #2 and #3 showed one band of the expected size of 2.2 Kb. (C) Genomic DNA from WT, Δ*sgl1* and transformants #1, #2 and #3, was probed with the *GMT1* gene probe, showing a 3.8 Kb band correspondent to *GMT1* gene in the WT and Δ*sgl1.* No band was detected in transformant #1, #2 and #3. Transformant #1 was named Δ*gmt1*/Δ*sgl1* mutant.

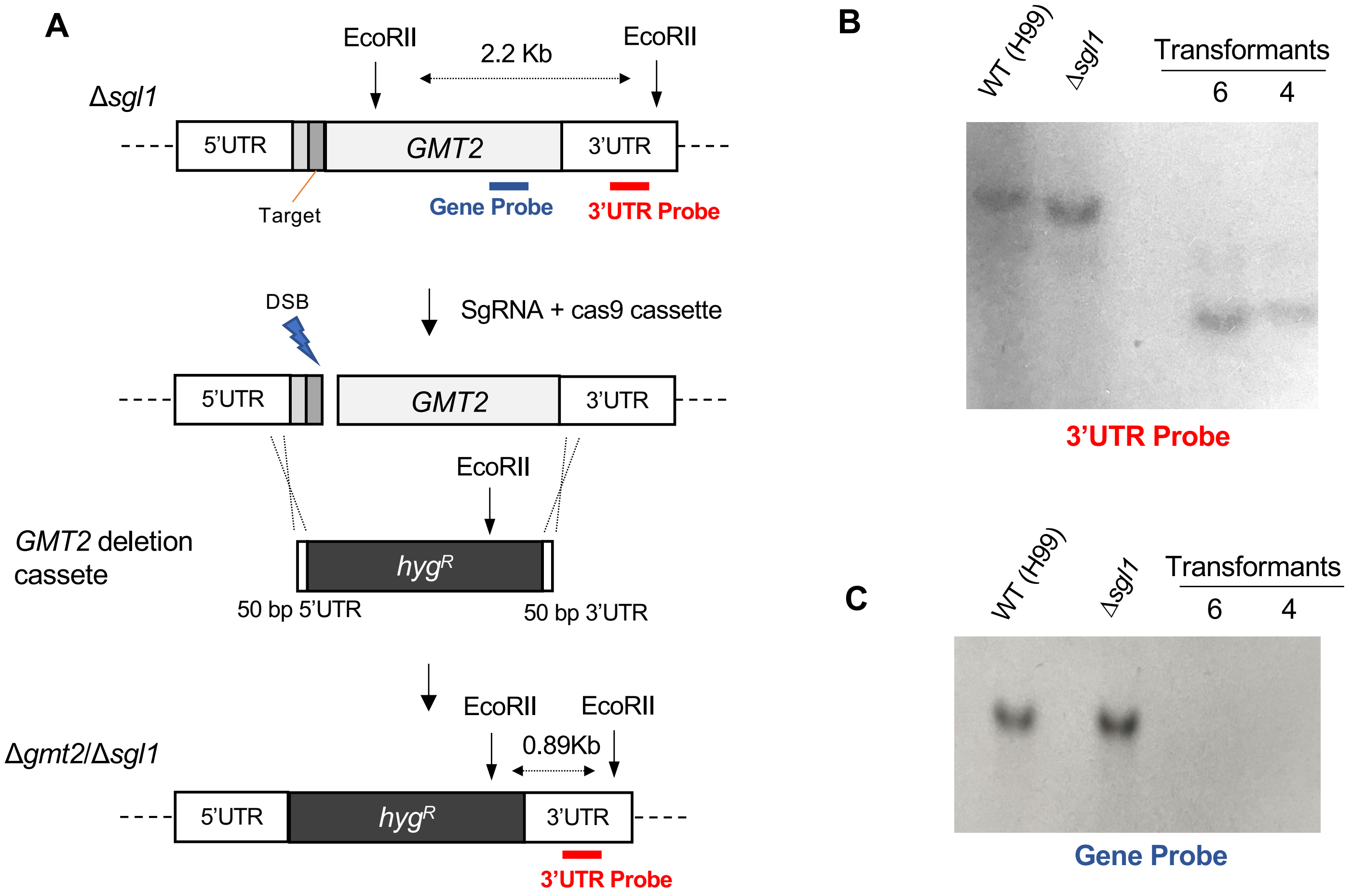

Supplementary Figure 3. Deletion of the *GMT2* gene in the *Δsgl1* background. (A) Diagram of the targeted deletion of *GMT2* gene via CRISPR-Cas9-mediated homologous recombination in the background of Δ*sgl1* strain. The Cas9 target site is exemplified in dark gray and the sites of homologous recombination with the selective marker are represented in white. (B) Transformants #4 and #6 were subjected to Southern Blot analysis. Genomic DNA was digested with EcoRII restriction enzyme and then incubated with 3`-UTR probe. The control WT (H99) and Δ*sgl1* showed a band of 2.2 Kb, whereas transformant #4 and #6 has one band of the expected size of 0.89 Kb. (C) Genomic DNA was probed with the *GMT2* gene probe, showing a 3.8 Kb band corresponding to *GMT2* gene in the WT and Δ*sgl1.* No band was detected in transformant #4 and #6. Transformant 6 was named Δ*gmt2*/Δ*sgl1* mutant.

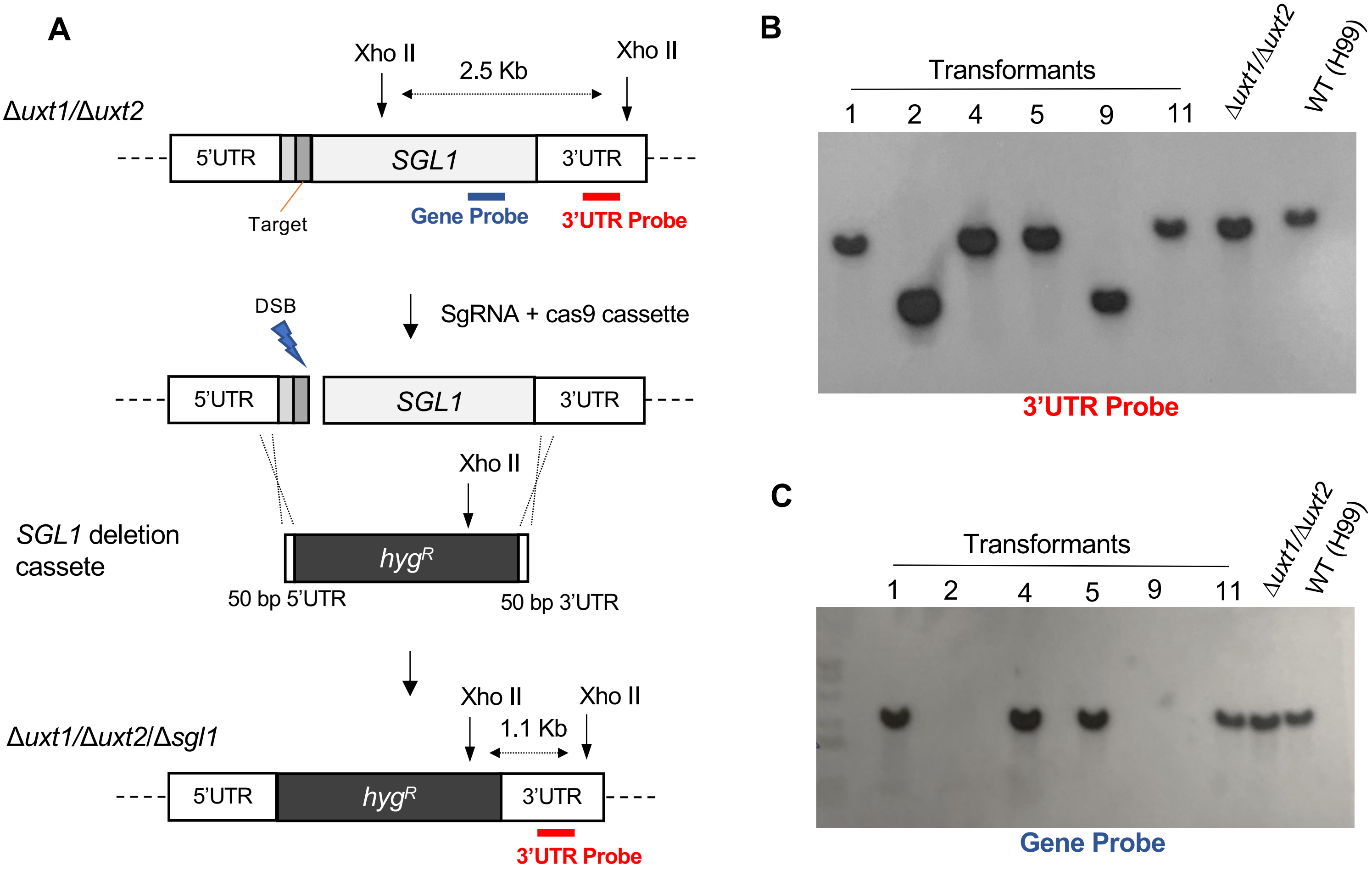

Supplementary Figure 4. Deletion of the *SGL1* gene in the *Δuxt1/Δuxt2* background. (A) Diagram of the targeted deletion of *SGL1* via CRISPR-Cas9-mediated homologous recombination in the background of *Δuxt1/Δuxt2*. The Cas9 target site is exemplified in dark gray and the sites of homologous recombination with the selective marker are represented in white. (B) The transformants were subjected to Southern Blot analysis. Genomic DNA was digested with XhoII restriction enzyme and then incubated with 3`-UTR probe. The control WT (H99) and *Δuxt1/Δuxt2* showed an expected band of 2.5Kb, and transformant #2 and #9 showed one expected band of 1.1 Kb. (C) Genomic DNA was probed with the *SGL1* gene probe, showing a 2.2 Kb band corresponding to the *SGL1* gene in the WT and *Δuxt1/Δuxt2.* Transformant #2 and #9 showed no band. Transformant #2 was named *Δuxt1/Δuxt2/Δsgl1* mutant.

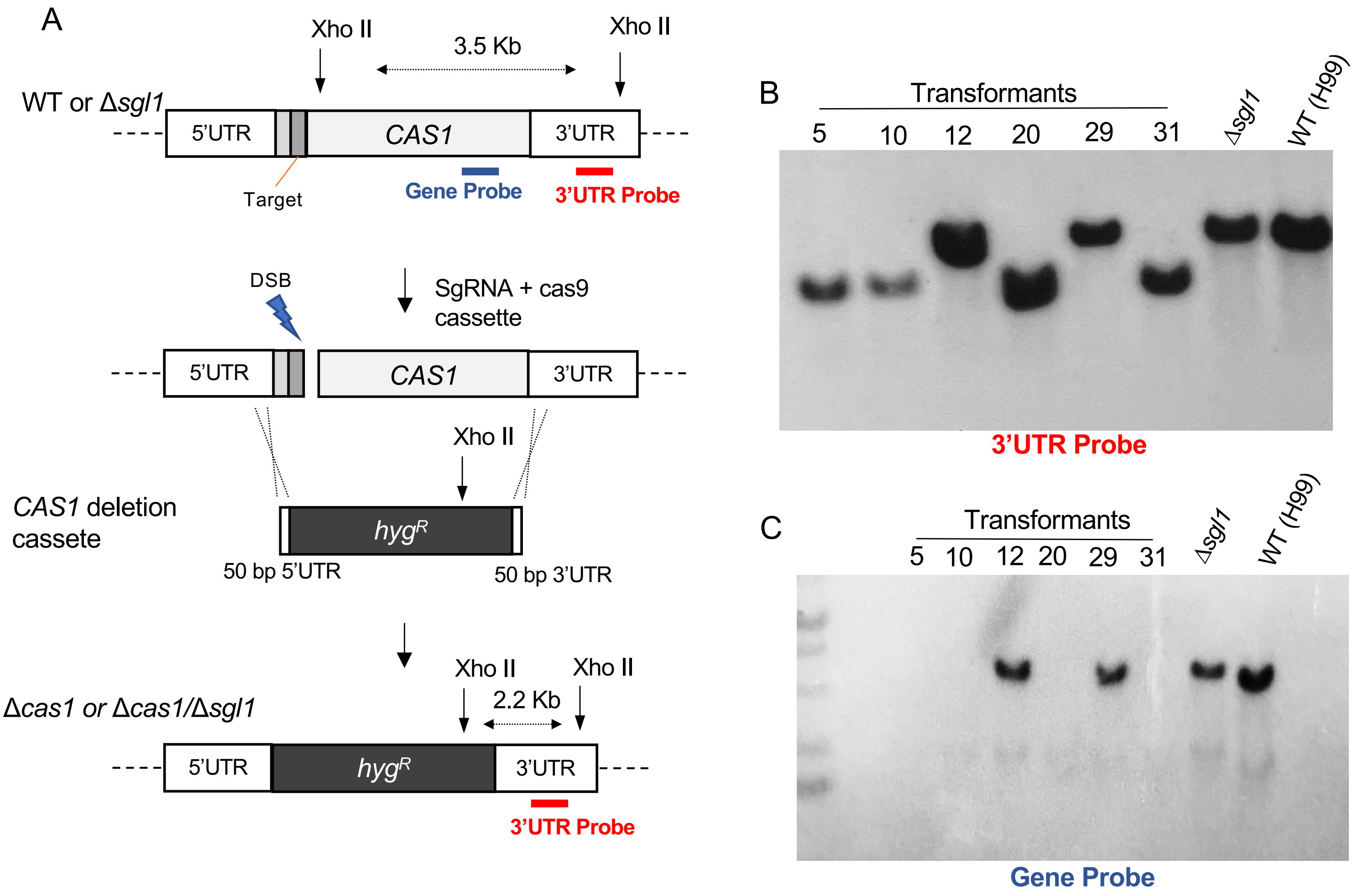

Supplementary Figure 5. Deletion of the *CAS1* gene in the Δ*sgl1* background. (A) Diagram of the targeted deletion of *CAS1* gene via CRISPR-Cas9-mediated homologous recombination in the background of Δ*sgl1* strain. The Cas9 target site is exemplified in dark gray and the sites of homologous recombination with the selective marker are represented in white. (B) The transformants were subjected to Southern Blot analysis. Genomic DNA was digested with XhoII restriction enzyme and then incubated with 3`-UTR probe. The control WT (H99) and Δ*sgl1* showed an expected band of 3.5 Kb, whereas transformants #5, #12, #20 and #31 showed an expected band of 2.2 Kb. (C) Genomic DNA was probed with the *CAS1* gene probe, showing a 2.2 Kb band correspondent to *CAS1* gene in the WT and Δ*sgl1*, whereas no band was detected in transformant #5, #20 and #31. Transformant #5 was named Δ*cas1*/Δ*sgl1* mutant.

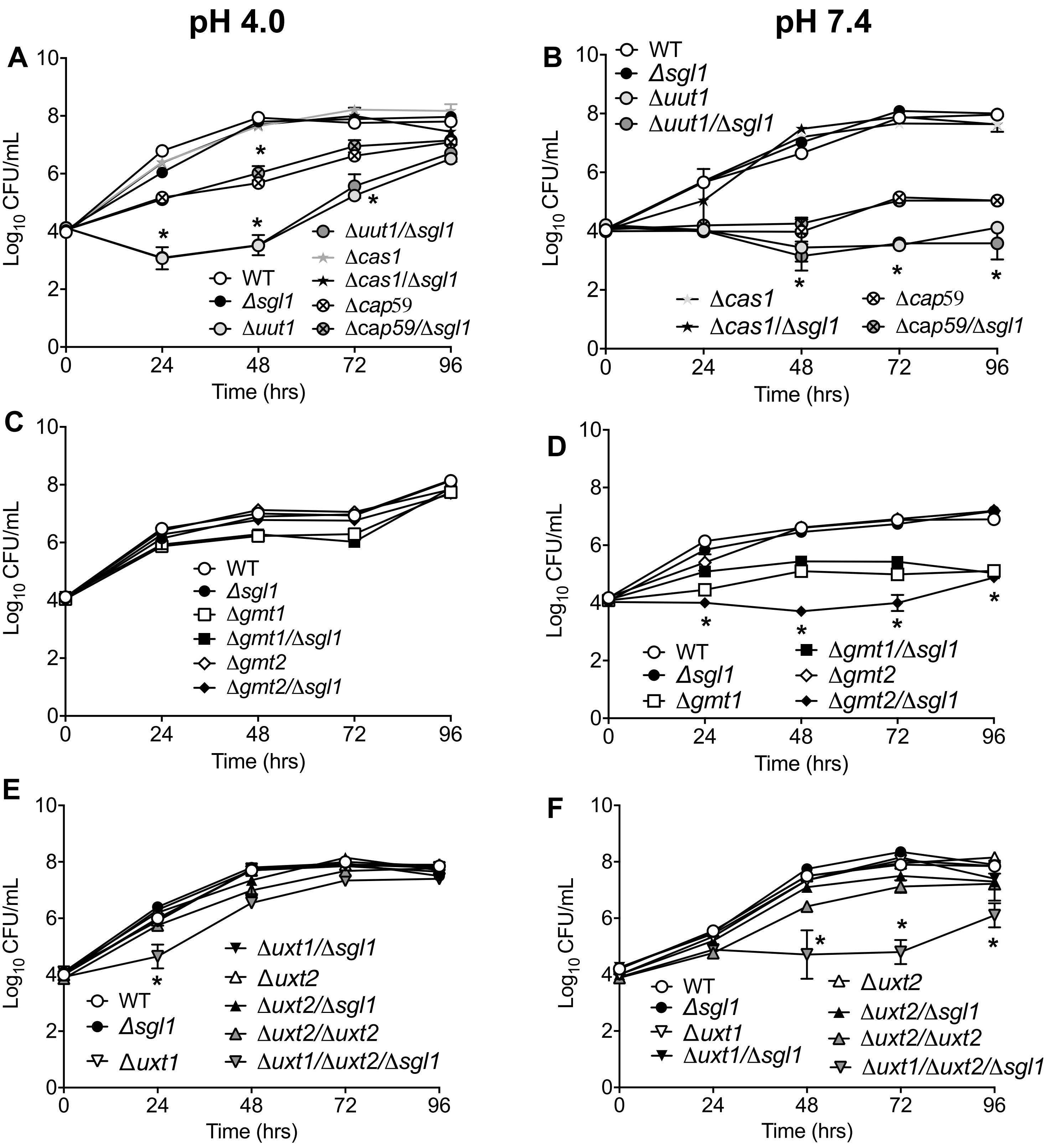

Supplementary Figure 6. Growth curves. Fungal cells were cultivated in physiological medium: DMEM plus 5% CO_2 at 37°C, at pH 4.0 (A, C, E) and pH 7.4 (B, D, F). A significant growth defect was observed in Δ*uut*1 at pH 4.0 when compared to the wild type or to the Δ*sgl1*. *, $P<0.05$ versus wild-type. An impairment in cell growth at pH7.4 was observed for Δ*uut1*, Δ*uut1*/Δ*sgl1*, Δ*gmt1*, Δ*gmt1*/Δ*sgl1* and, Δ*gmt2*/Δ*sgl1* when compared to WT. *, $P<0.05$ versus wild-type. Values are expressed as mean ± standard deviation from three independent experiments (n=3) and analyzed by ANOVA two-way followed by Tukey post-test.

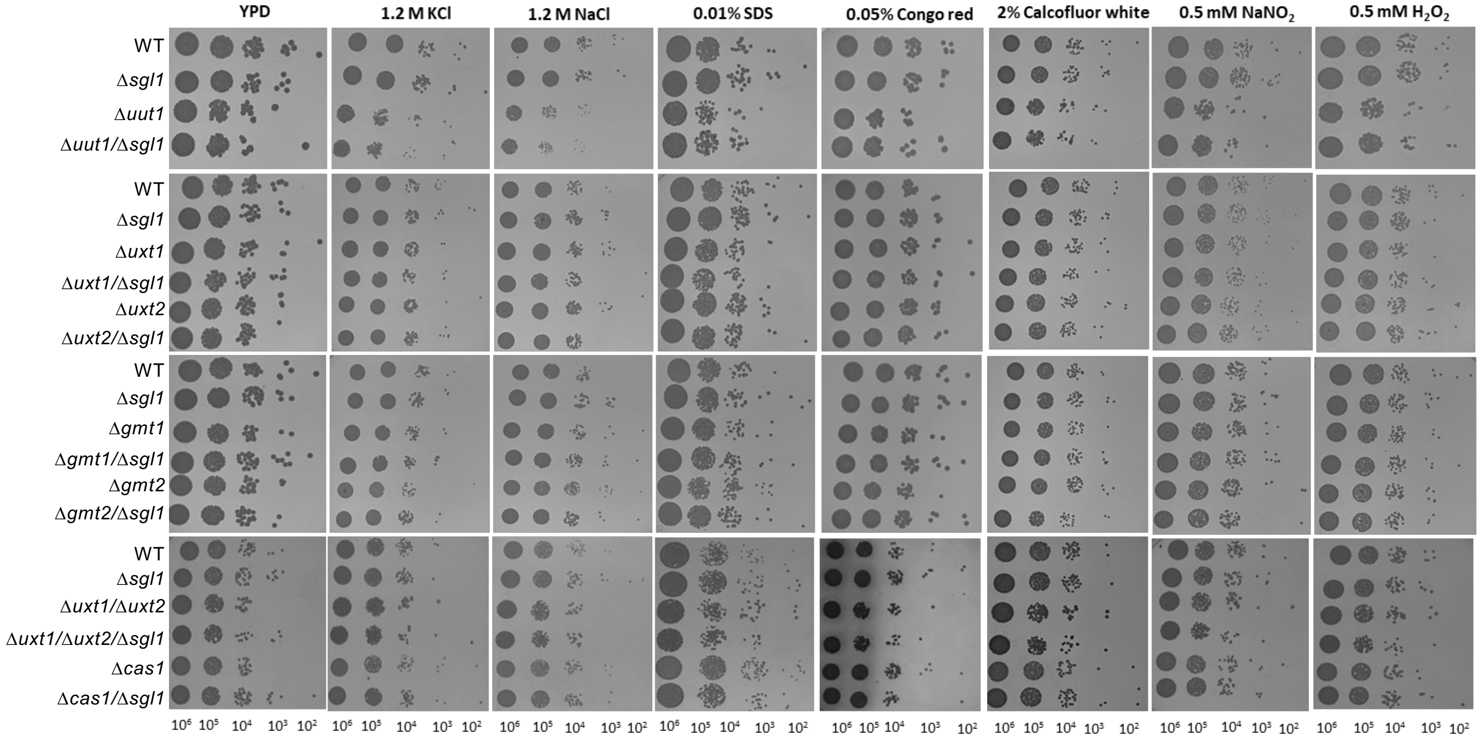

Supplementary Figure 7. Effect of stress growing conditions on mutant strains. The stress conditions tested included YPD containing 0.01% SDS, 1.2 M NaCl, 1.2 M KCl, 0.05% Congo Red (CR), or 2% Calcofluor White (CFW). To test oxidative and nitrosative stress sensitivity, dilutions were spotted onto solid YPD medium (supplemented with 0.5 mM hydrogen peroxide (H_2O_2) or 0.5 mM sodium nitrite ($NaNO_2$).

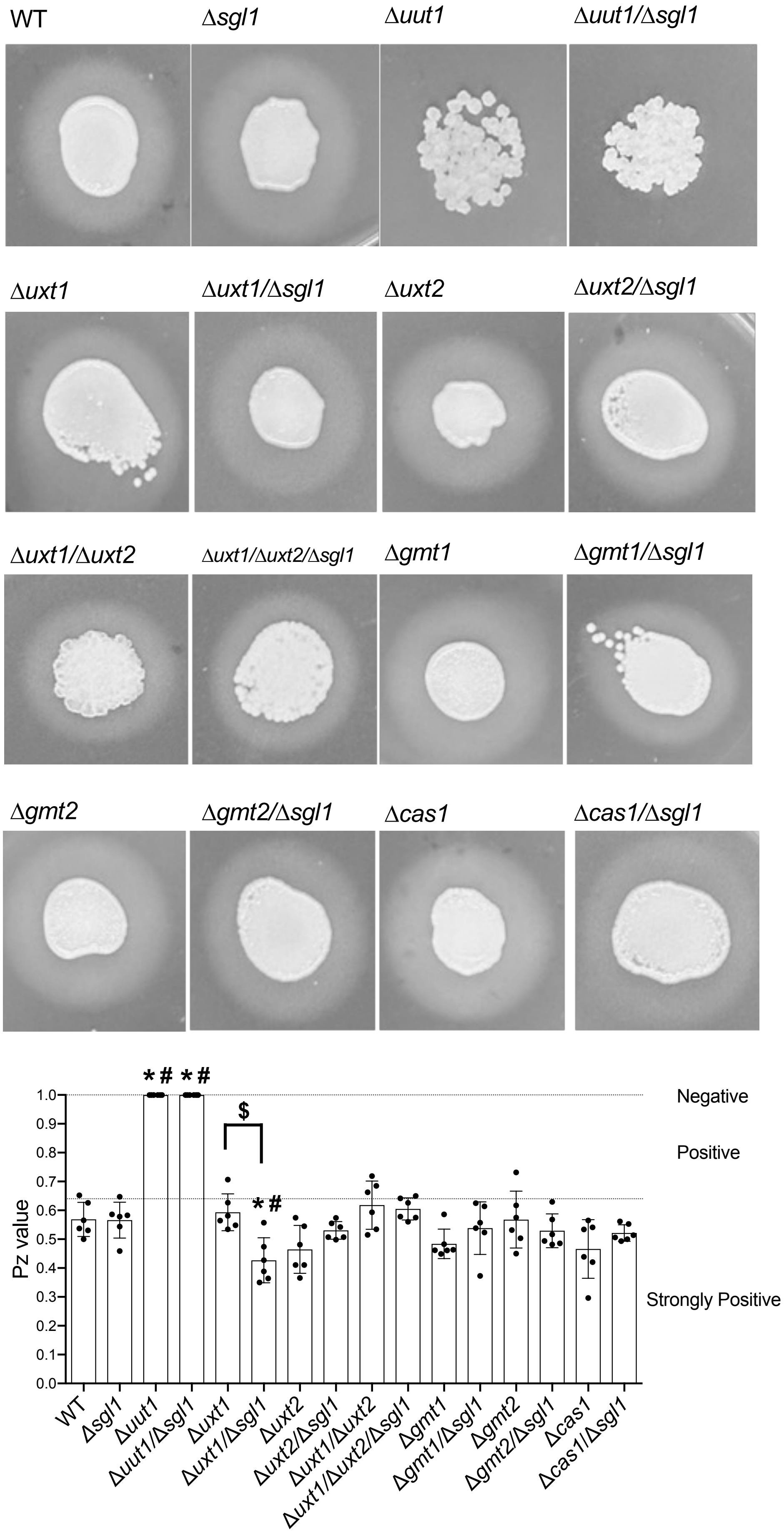

Supplementary Figure 8. Measurement of phospholipase activity. *C. neoformans* strains were screened for extracellular phospholipase production. Cultures were incubated at 37°C in egg yolk media, and the diameter of the zone of precipitate around the colonies was measured after 5 days. The ratio of the diameter of the colony to the total diameter of the colony plus precipitation zone (Pz) was measured as an index of phospholipase activity. A Pz value of 1.0 as observed in Δ*uut1* and Δ*uut1*/Δ*sgl1* indicating that these strains are negative for phospholipase activity. The Δ*uxt1*/Δ*sgl1* mutant has higher phospholipase activity when compared to wild type, (*, $P<0.05$), Δ*sgl1*, ($^{\#}$, $P<0.05$), or Δ*uxt1* ($^{\$}$, $P<0.05$). Values are expressed as mean ± standard deviation from six independent experiments (n=6) and analyzed by one-way ANOVA followed by Tukey's post-test.

A

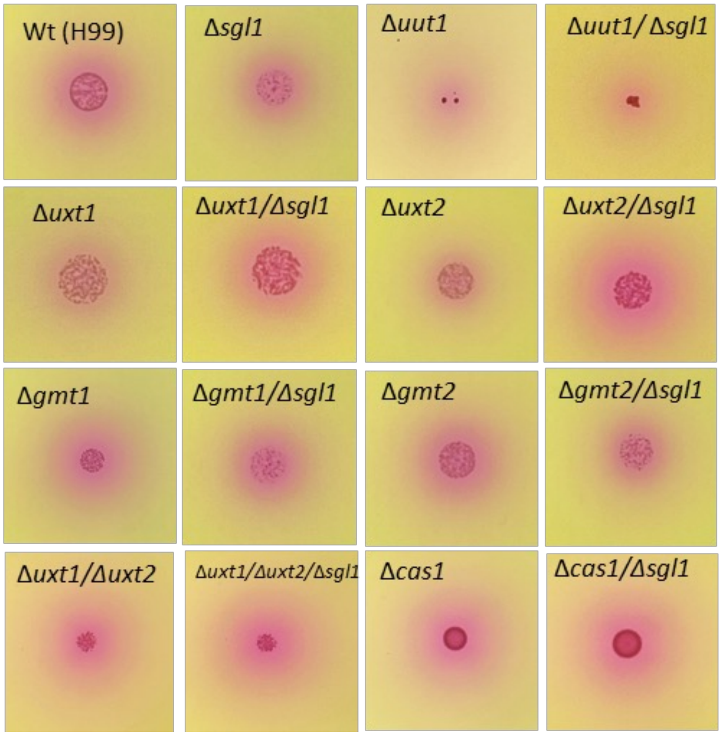

B

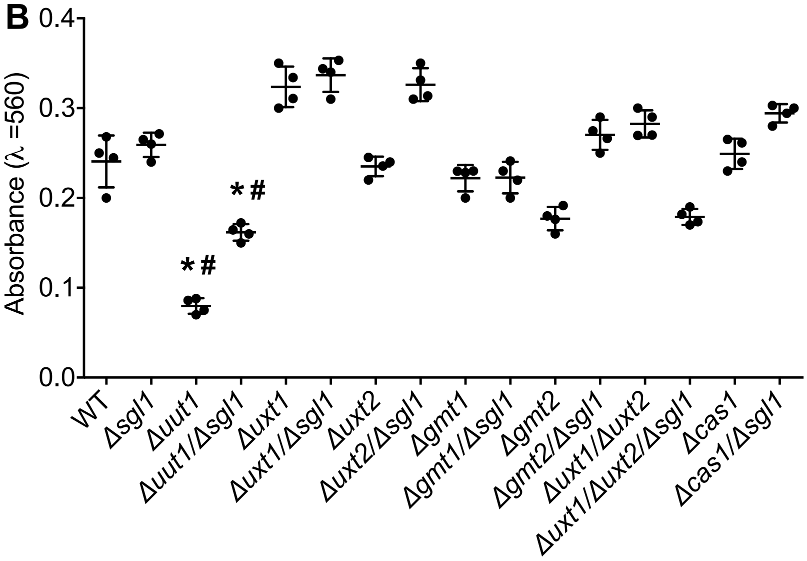

C

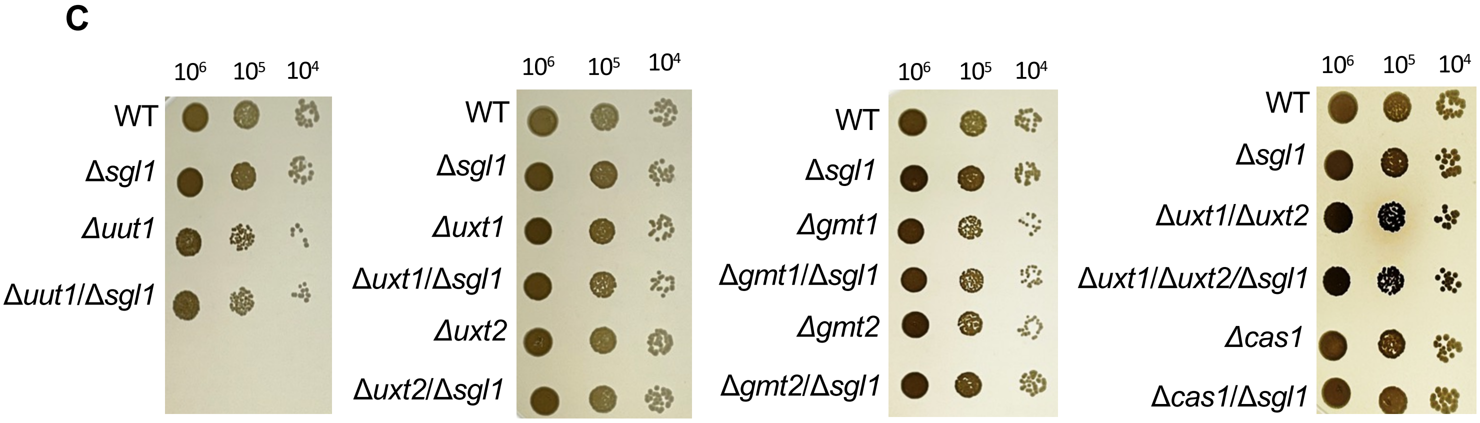

Supplementary Figure 9. Measurement of urease activity and melanin production. **A)** The strains were grown in Christensen's Urea Agar for 5 days and then observed for the pink pigment formation. **B)** In order to quantify the urease activity, the yeasts were grown in Urea Broth, and after 4 hours of incubation the absorbance at 560 nm was measured. Values are expressed as mean ± standard deviation from three independent experiments (n=3) and analyzed by one-way ANOVA followed by Tukey's post-test. *, $P<0.05$ versus WT; $^{\#}$, $P<0.05$ versus Δ*sgl1*. **C)** To assess cell-associated melanin production, 5 µL of a 10^6 - 10^4 cells/mL solution was plated on agar plates containing 8 mg/mL KH_2PO_4, 2 mg/mL glucose, 2 mg/mL L-glycine, 1 µg/mL D-biotin, 1µg/mL thiamine, 0.92 mg/mL $MgSO_4$ $7H_2O$, and 0.4 mg/mL L-3,4-dihydrohyphenylalanine (L-DOPA; Sigma Aldrich). All cells tested showed pigment compatible with melanin production. Data are representative of 3 separate experiments.

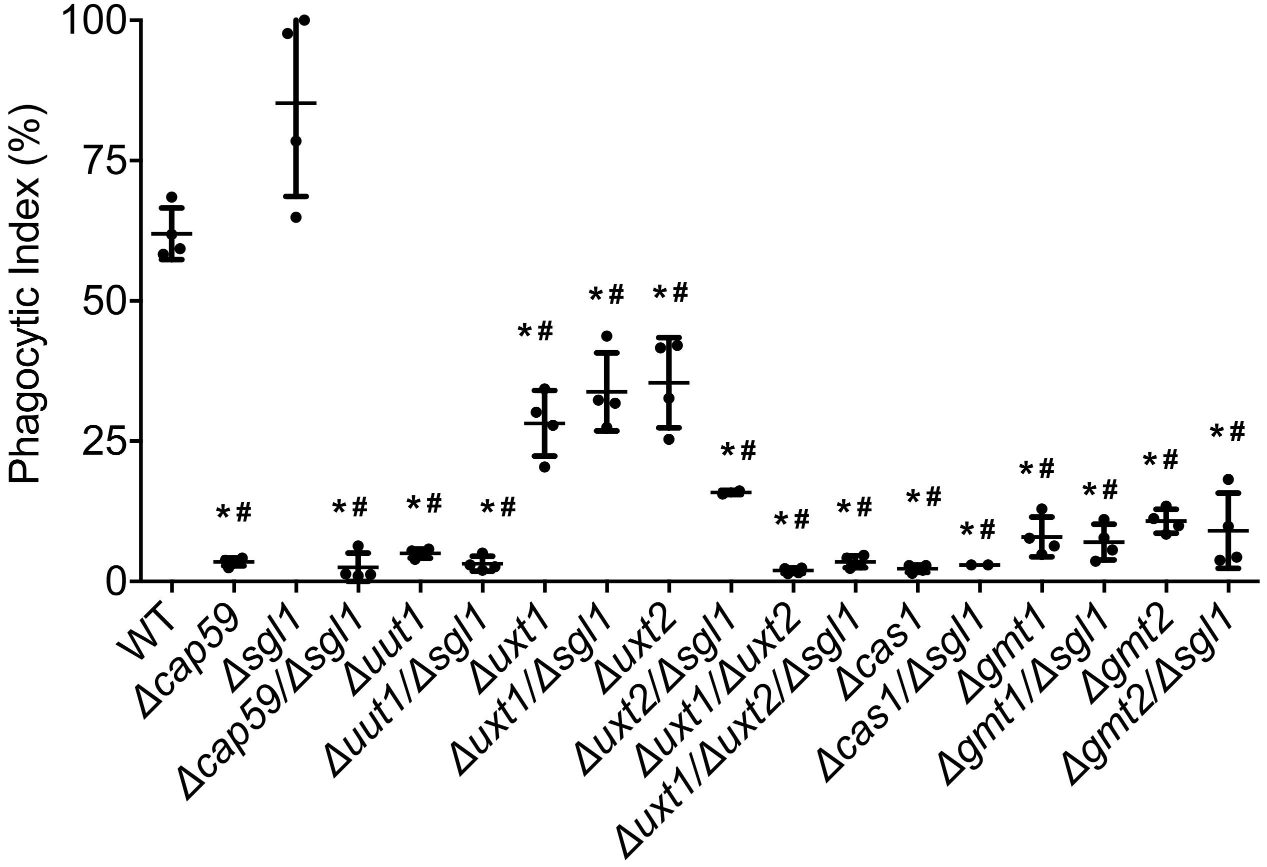

Supplementary Figure 10. Assessment of phagocytosis of the mutant strains. Phagocytic index was defined as the percentage of yeast cells attached or ingested per number of macrophages per field. *, *P<0.05* versus the WT strain; #, *P*<0.05 versus the Δ*sgl1*. Values are expressed as mean ± standard deviation from three independent experiments (n=3) and analyzed by one-way ANOVA followed by Tukey's post-test.

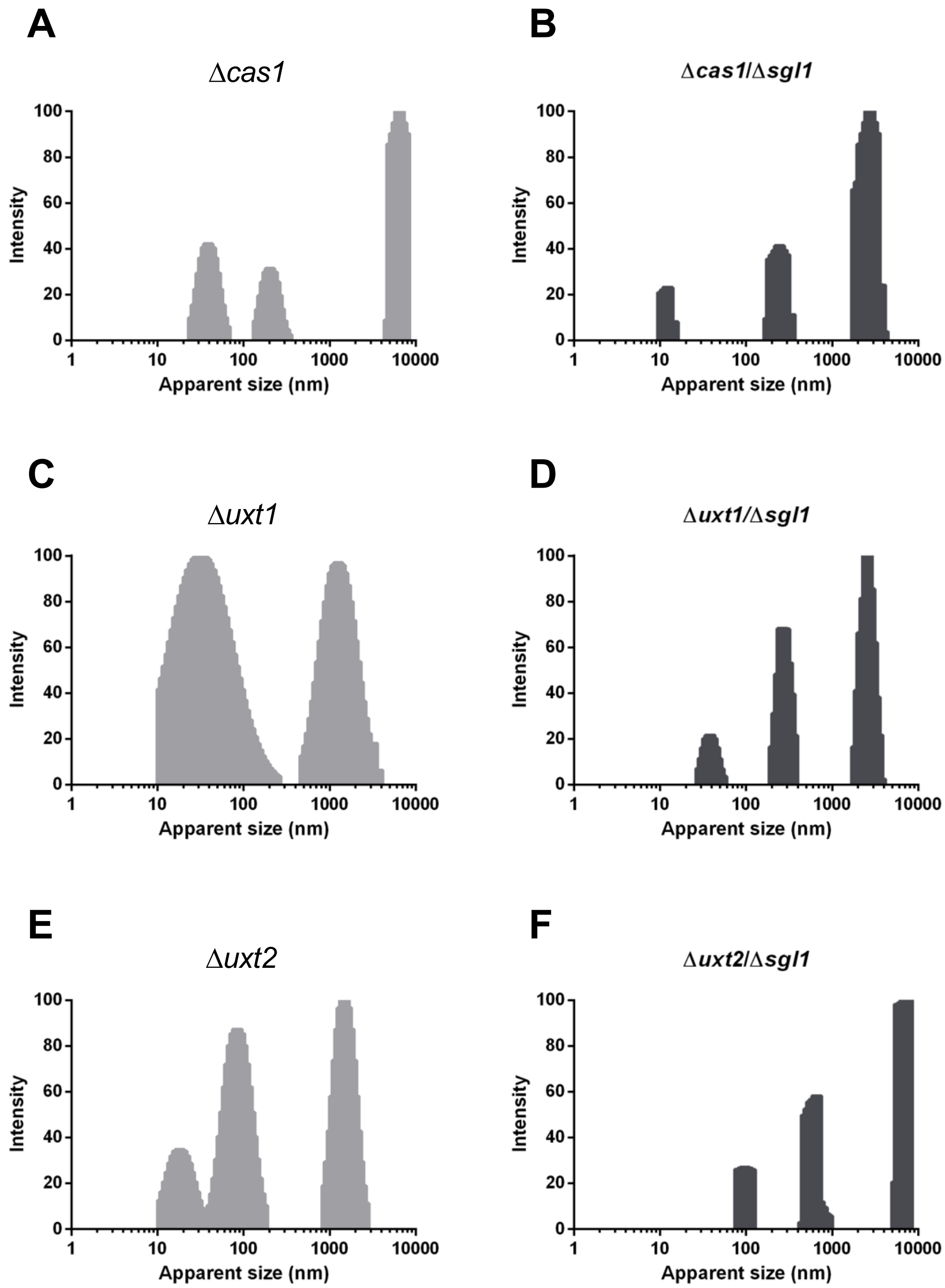

Supplementary Figure 11. Size distribution of capsular GXM. The size distribution of GXM from *C. neoformans* Δ*cas1*, Δ*cas1*/Δ*sgl1*, Δ*uxt1*, Δ*uxt1*/Δ*sgl1*, Δ*uxt2*, *and* Δ*uxt2*/Δ*sgl1* was analyzed using dynamic light scattering (DLS). The x-axis represents the measured particle size distribution, while the y-axis corresponds to the intensity-weighted size percentages.

Glycosyl residues	WT Mol %	Δ*sgl1* Mol %	Δ*gmt1*/Δ*sgl1* Mol %	Δ*gmt2*/Δ*sgl1* Mol %
Xylose	29.8	28.0	30.7	22.8
Glucuronic acid	8.8	9.7	7.2	9.0
Mannose	61.4	62.3	62.1	68.2
Sum	100	100	100	100

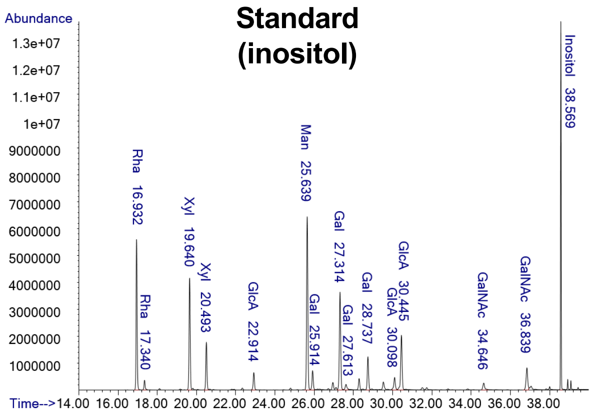

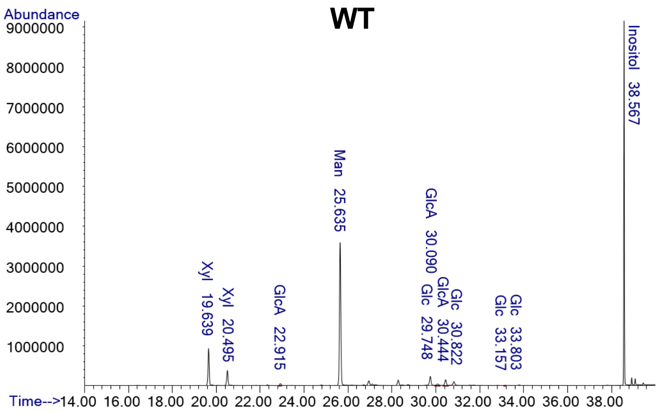

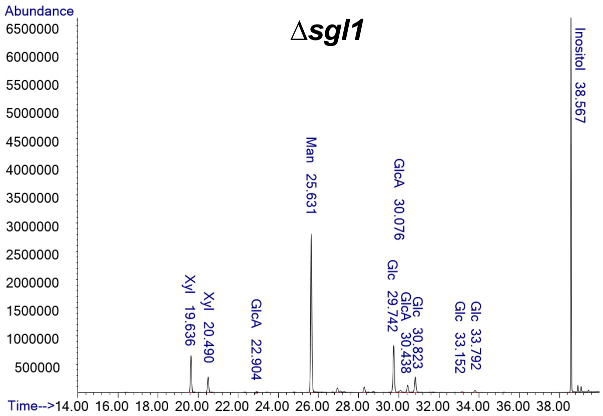

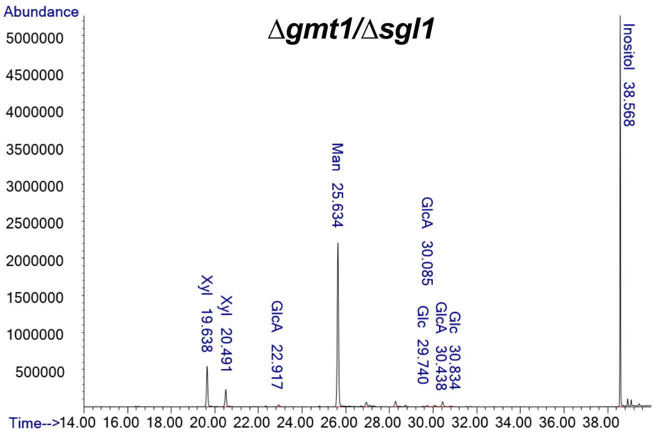

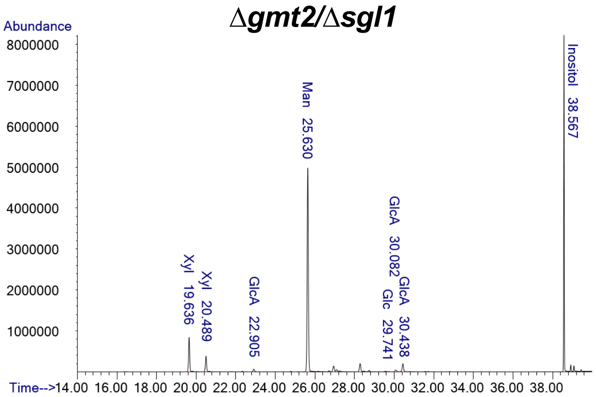

Supplementary Figure 12. Analysis of GXM by trimethylsilyl (TMS) derivatization. Calculated Mol % of monosaccharides without glucose by GC-MS for GXM isolated from *C. neoformans* wild-type, Δ*sgl1*, Δ*gmt1*/Δ*sgl1*, and Δ*gmt2*/Δ*sgl1*. The GXM from these samples are mainly composed of xylose, glucuronic acid and mannose, which is consistent with the known structure of GXM. No significant alterations of glycosyl composition of GXM from Δ*gmt1*/Δ*sgl1* and Δ*gmt2*/Δ*sgl1* were observed compared to WT and Δ*sgl1*.
